# Targeting Mesothelin Enhances Personalized Neoantigen Vaccine Induced Antitumor Immune Response in Orthotopic Pancreatic Cancer Mouse Models

**DOI:** 10.1002/advs.202407976

**Published:** 2025-01-31

**Authors:** Zhixiong Cai, Zhenli Li, Wenting Zhong, Fangzhou Lin, Xiuqing Dong, Honghao Ye, Yutong Guo, Geng Chen, Xiaoling Yu, Haijun Yu, Ruijing Tang, Xiaolong Liu

**Affiliations:** ^1^ The United Innovation of Mengchao Hepatobiliary Technology Key Laboratory of Fujian Province Mengchao Hepatobiliary Hospital of Fujian Medical University Fuzhou 350025 P. R. China; ^2^ The Liver Center of Fujian Province Fujian Medical University Fuzhou 350025 P. R. China; ^3^ Mengchao Med‐X Center Fuzhou University Fuzhou 350116 P. R. China; ^4^ State Key Laboratory of Drug Research & Center of Pharmaceutics Shanghai Institute of Materia Medica Chinese Academy of Sciences Shanghai 201203 P. R. China

**Keywords:** apCAFs, immunotherapy, mesothelin, neoantigen vaccine, pancreatic cancer

## Abstract

The immunosuppressive microenvironment in pancreatic cancer, characterized by low tumor‐specific T cells and excessive fibrosis, limits the effectiveness of immunotherapy. Here, three datasets and multi‐immunofluorescence staining of tissue microarrays in pancreatic cancer indicate that mesothelin (MSLN) expression negatively correlates with cytotoxic T cells in tumor. Anti‐MSLN antibody (*α*MSLN) treatment of pancreatic cancer in vivo can significantly increase T cell infiltration. Meanwhile, the combination of *α*MSLN and neoantigen peptide vaccine identified from pancreatic cancer cell lines is demonstrated to be more effective in inducing neoantigen‐specific T cell generation and infiltration at subcutaneous and orthotopic pancreatic cancer models for enhancing antitumor efficacy. Single‐cell transcriptome analysis shows that the combined treatment significantly reduces the proportion of fibroblasts, improves the infiltration of IFN‐*γ* ^+^CD4^+^ and GZMK^+^CD8^+^ T cells, as well as reduces the interaction of antigen presentation‐associated ligands and receptors between antigen‐presenting Cancer‐Associated Fibroblasts (apCAFs) and naive CD4^+^ T cells. The negative correlations between apCAFs and CD8^+^ T cells/IFN‐*γ* ^+^CD4^+^ T cells are further confirmed in human pancreatic cancer tissues. Overall, this study demonstrates that targeting MSLN can improve neoantigen vaccine induced immune efficacy by reducing apCAFs to interrupt the conversion of naive CD4^+^ T cells to Tregs, and therefore increase the infiltration of tumor‐specific T cells.

## Graphical abstract

Targeting mesothelin (MSLN) improved neoantigen vaccine efficacy in pancreatic cancer by reducing apCAFs, which disrupted the conversion of naive CD4^+^ T cells into Tregs and enhanced the infiltration of IFN‐*γ* ^+^CD4^+^ and GZMK^+^CD8^+^ T cells. This combination strengthened neoantigen‐specific T cell response to overcome the immunosuppressive tumor microenvironment, offering a promising strategy to enhance immunotherapy outcomes in pancreatic cancer.

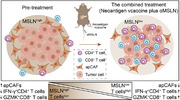

## 1 Introduction

Pancreatic cancer is characterized as a highly malignant gastrointestinal tumor with insidious onset and rapid progression, and the 5‐year survival rate of patients is less than 9%.^[^ ^1^ ^]^ The main treatment modality for early‐onset pancreatic cancer is still radical surgery; however, due to the difficulty of early diagnosis, only 15–20% of patients are eligible for surgical resection. Single‐agent or combination therapy based on gemcitabine is the foundation of treating advanced pancreatic cancer, but the overall response rate is less than 20%.^[^ ^2^ ^]^ Recently, immunotherapies provided new opportunities for pancreatic cancer treatment. However, immunotherapy based on PD‐L1 and CTLA‐4 immune checkpoint inhibitors, has shown limited effectiveness in pancreatic cancer patients, with only 3.1% achieving objective responses.^[^ ^3^ ^]^ This is mainly because pancreatic cancer is mostly considered as a “cold” tumor with limited immune cell infiltration. Therefore, how to generate tumor specific immune cells and enhance their infiltration are the key factors for improving the immunotherapy efficacy of advanced pancreatic cancer.

Neoantigens, belonging to tumor‐specific antigens, are peptides or proteins produced by tumor cells due to genetic variation (including tumor specific mutation/indel/RNA editing). Some epitopes of these neoantigens can be recognized by the immune system, leading to the generation of tumor antigen‐specific memory T cells, and some of which can infiltrate into the tumor site and attack tumor cells accurately. Studies have shown that a higher number of neoantigens with stronger immunogenicity in pancreatic cancer patients are associated with better prognosis; furthermore, patients with long‐term survival (over 5 years) have 12 times more activated CD8^+^ T cells targeting neoantigens in their primary tumors compared to those with short‐term survival.^[^ ^4^ ^]^ Significantly, a recent phase 1 clinical trial demonstrated that the neoantigen vaccine Autogene cevumeran (BNT122), in combination with Atezolizumab (anti‐PD‐L1 monoclonal antibody) and mFOLFIRINOX, induced a substantial tumor‐specific T cell response in surgically resected pancreatic cancer patients associated with delayed relapse.^[^ ^5^ ^]^ However, the complex tumor immunosuppressive microenvironment in advanced pancreatic cancer often leads to the inability of generated tumor‐specific T cells to effectively enter into tumor or the easy dysfunction of those already entering the tumor, resulting in limited antitumor efficacy. Therefore, further improving the infiltration and function of tumor‐specific T cells in pancreatic cancer is the key to improve the therapeutic efficacy of neoantigen vaccines and still need to be explored.

Mesothelin (MSLN), a 40‐kDa glycosylphosphatidyl inositol‐linked protein, is highly expressed in many solid tumors, including pancreatic cancer (80‐85%), while its expression is limited in normal tissues. Therefore, MSLN has become an attractive target for tumor‐specific therapy. Currently, the treatment of tumors targeting MSLN includes antibody drugs and CAR‐T cells, which have shown certain antitumor efficacy in clinical trials.^[^ ^6^ ^]^ Monoclonal antibody targeting MSLN, such as Amatuximab (MORAb‐009), could disrupt cell adhesion and trigger antibody‐dependent cell cytotoxicity, leading to the destruction of MSLN‐expressing cells.^[^ ^7^ ^]^ Primarily, MSLN antibodies exert their effects through antibody‐dependent cellular cytotoxicity (ADCC), where they bind to MSLN on target cells and recruit natural killer (NK) cells via their Fc regions. These NK cells release cytotoxic granules, inducing apoptosis in MSLN‐expressing cells. Additionally, MSLN antibodies can activate complement‐dependent cytotoxicity (CDC), triggering the complement cascade, which results in the formation of membrane attack complexes and subsequent cell lysis. Furthermore, MSLN antibodies exhibit direct anti‐proliferative effects by disrupting MSLN‐mediated signaling pathways or inducing antibody‐dependent cellular phagocytosis (ADCP). Animal studies also show that MSLN antibody treatment increases the sensitivity to gemcitabine, reduces the expression of c‐Met and AKT in the liver, and concurrently decreases the migratory capacity of pancreatic cancer cells.^[^ ^8^ ^]^ Recent study also suggest that anti‐MSLN antibody can effectively inhibit the transformation of mesothelial cells into fibroblasts, significantly reduce the ratio of regulatory T cells (Tregs)/CD8^+^ T cells, to inhibit pancreatic cancer progression.^[^ ^9^ ^]^ This implies that anti‐MSLN antibody might serve as an immunomodulator for pancreatic cancer, but further investigation is still needed.

**Scheme 1:**
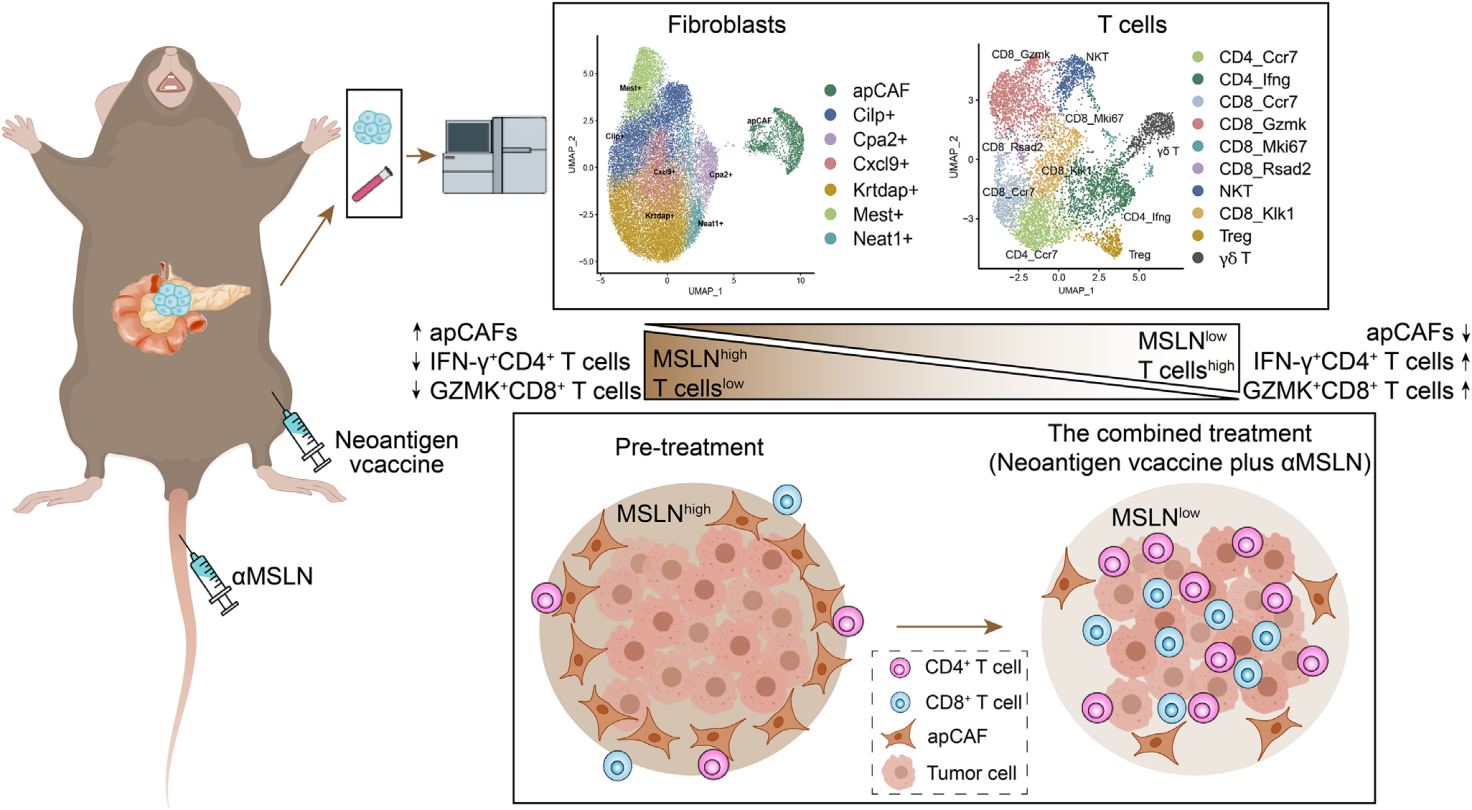
Schematic illustration of the modulation of the tumor microenvironment by the combined treatment of the personalized neoantigen vaccine and anti‐MSLN antibody (*α*MSLN). Single‐cell transcriptome analysis reveals that the combined treatment reduces fibroblasts and enhances IFN‐*γ* ^+^CD4^+^ and GZMK^+^CD8^+^ T cell infiltration in pancreatic cancer tissues, ultimately improving antitumor efficacy.

Here, we evaluated the relationship between MSLN expression and cytotoxic CD8^+^ T cell infiltration in pancreatic cancer, and investigated the feasibility and safety of combing personalized neoantigen vaccine with anti‐MSLN antibody to improve the immunotherapy efficacy of pancreatic cancer in subcutaneous and orthotopic mouse models. Furthermore, single‐cell sequencing and multi‐immunofluorescent histochemical staining was employed to deeply analyze the distribution and functional status of tumor‐infiltrating lymphocyte subpopulations and fibroblasts in the pancreatic cancer immune microenvironment after the combined treatment (**Scheme** **1**). These results would provide solid evidence for combing personalized neoantigen vaccine with anti‐MSLN antibody to treat pancreatic cancer.

## 2 Results

### 2.1 MSLN Expression is Negatively Correlated with CD3^+^CD8^+^ T Cell Infiltration in Pancreatic Cancer

**Figure 1:**
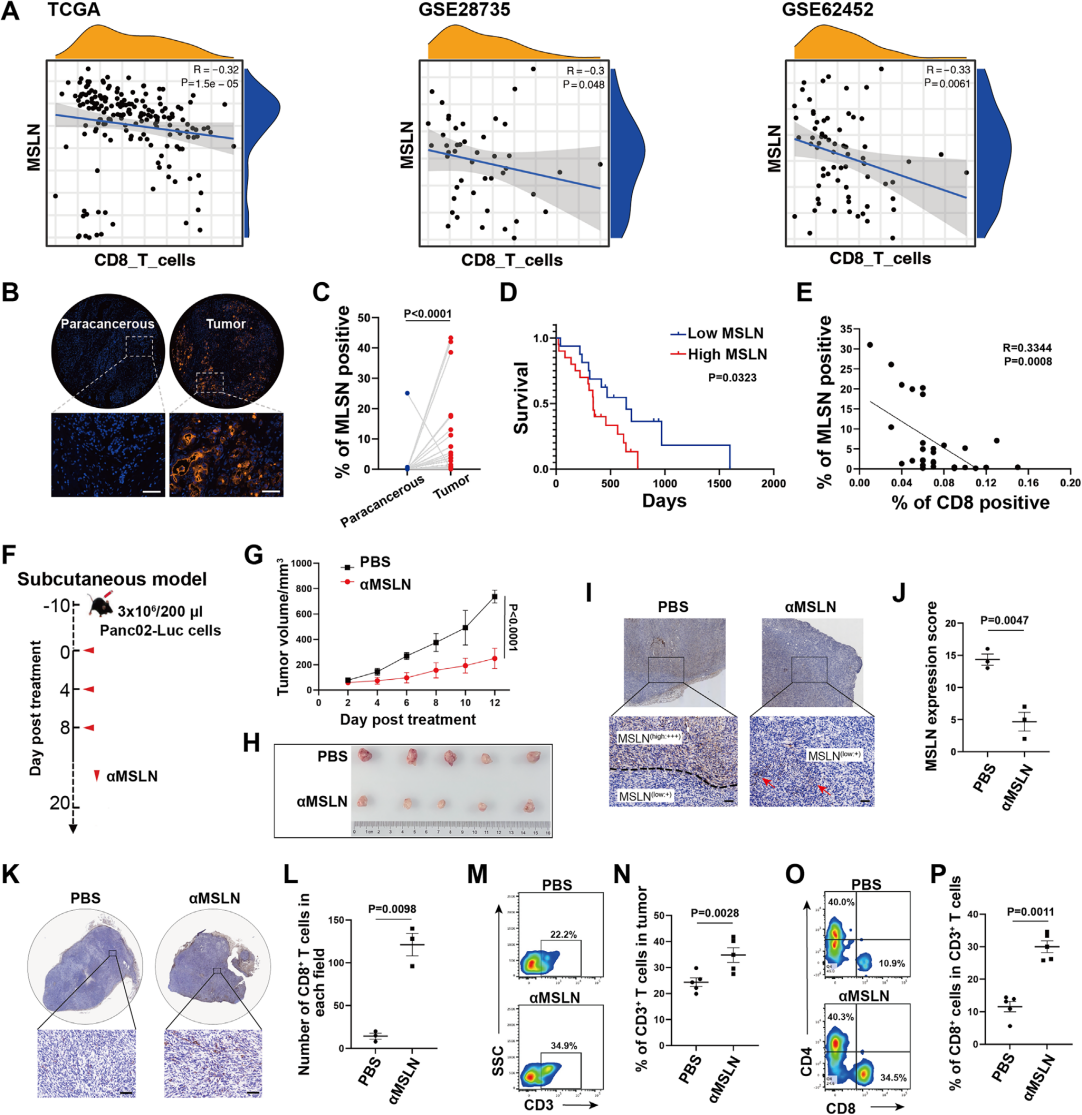
The correlation between MSLN expression and CD3^+^CD8^+^ T cell infiltration in pancreatic cancer. A) The correlation analysis between MSLN and CD3^+^CD8^+^ T cells in 3 datasets as indicated. B,C) The represent graph and the comparation of MSLN expression between pancreatic cancer and paracancerous tissues by tissue microarray with 30 cases of pancreatic cancer (blue: DAPI, orange: MSLN; n = 30; one‐way ANOVA). Scale bar = 50 µm. D) The association between MSLN expression and patient's survival. E) The linear correlation analysis between MSLN expression and CD3^+^CD8^+^ T cell infiltration in tissue microarray. F) Schematic diagram showing the timeline of subcutaneous pancreatic cancer model construction and treatment. G,H) Tumor volume monitoring in Panc02 tumor‐bearing C57BL/6 mice after receiving PBS and *α*MSLN treatment for three times, respectively (n = 5; two‐way ANOVA). I, J) The represent graph and statistic analysis of MSLN expression in Panc02 tumor after treatment by IHC staining (n = 3; one‐way ANOVA). Scale bar = 100 µm. K,L) The represent graph and statistic analysis of inflating CD3^+^CD8^+^ T cells in Panc02 tumor after treatment by IHC staining (n = 3; one‐way ANOVA). Scale bar = 100 µm. M‐P) The percentage and statistic analysis of CD3^+^, CD4^+^, and CD8^+^ T cells detected in Panc02 tumor after treatment (n = 5; one‐way ANOVA) by flow cytometry. Data are presented as the mean ± SEM. *p < 0.05, **p < 0.01, ***p < 0.001, ****p < 0.0001; ns, no significance.

There is debate about whether the expression of MSLN is related to T cell infiltration in pancreatic cancer.^[^ ^10^ ^]^ Here, we first focused on the correlation between MSLN expression and the infiltration of cytotoxic CD3^+^CD8^+^ T cells in clinical samples of pancreatic cancer. Utilizing tumor transcriptomic data from 183 pancreatic cancer patients in The Cancer Genome Atlas (TCGA), we analyzed the correlation between MSLN expression and the gene signature of CD3^+^CD8^+^ T cell population. As shown in **Figure** **1**A, linear fitting curve indicated a significant negative correlation between MSLN expression and CD3^+^CD8^+^ T cells (p = 1.5e‐05). This finding was further validated using another two pancreatic cancer transcriptomic datasets [GSE28735 (n = 45, p = 0.048) and GSE62452 (n = 69, p = 0.0061)] from the Gene Expression Omnibus (GEO) database. However, such correlation between MSLN expression and CD3^+^CD4^+^ T cells was not observed in those 3 datasets (p = 0.7, Figure S1, Supporting Information). Subsequently, we further validated the correlation between MSLN expression and CD3^+^CD8^+^ T cell infiltration in 30 paired pancreatic tumor tissues and adjacent tissues using multiple immunofluorescence technique on tissue microarrays. As illustrated in Figure 1B,C, MSLN expression in pancreatic cancer was significantly higher compared to adjacent tissues (*p* < 0.0001). Importantly, there was a significant negative correlation between MSLN expression and the infiltration of CD3^+^CD8^+^ T cells (p = 0.0008); and the patients with high MSLN expression in tumor tissue showed poorer 5‐years survival (p = 0.0323, Figure 1D,E). These results suggested that MSLN expression in pancreatic cancer might inhibit CD3^+^CD8^+^ T cell infiltration.

To further investigate whether targeting MSLN with antibody (*α*MSLN) could enhance CD3^+^CD8^+^ T cell infiltration in pancreatic cancer, a mouse subcutaneous pancreatic cancer model based on Panc02 cells (mouse pancreatic ductal adenocarcinoma cell line that mimics the growth characteristics and immune microenvironment of human pancreatic tumors) was established and treated with *α*MSLN (Figure 1F). As shown in Figure 1G,H, consistent with literature reports, *α*MSLN treatment could significantly inhibit pancreatic cancer growth (*p* < 0.0001). Then IHC staining demonstrated a significant reduction in MSLN expression (p = 0.0047) and a substantial increase in CD3^+^CD8^+^ T cell infiltration (p = 0.0098) in tumor tissue after *α*MSLN treatment, indicating that *α*MSLN can effectively kill the cells with high expression of MSLN and modulate the immune microenvironment in pancreatic cancer (Figure 1I–L). Flow cytometry analyzing the proportion of CD3^+^, CD4^+^ and CD8^+^ T cells in tumor tissue further confirmed that *α*MSLN treatment could significantly enhance the infiltration of CD3^+^CD8^+^ T cells (p = 0.0011) but not the CD3^+^CD4^+^ T cells (Figure 1M–P). This phenomenon was further validated in another subcutaneous pancreatic tumor model, established by inoculating a mouse pancreatic cancer KPC cell line (Pdx1‐cre/LSL‐KrasG12D/P53R172H) overexpressing chicken ovalbumin (OVA) antigen and luciferase(KPC‐OVA‐Luc) (Figure S2A, Supporting Information), whose pathological characteristics closely resemble those of human pancreatic cancer. The results showed that the tumor growth was significantly inhibited after *α*MSLN treatment (*p* < 0.0001), accompanied by significant infiltration of CD3^+^ and CD8^+^ T cells (*p* < 0.0001 and p = 0.0003, respectively, Figure S2B‐G, Supporting Information). To further validate the effect of MSLN overexpression on CD3^+^CD8^+^ T cell infiltration, Panc02 cells were transduced with lentivirus to overexpress MSLN, and then used to establish subcutaneous tumor model to evaluate T cell infiltration. The results showed that MSLN overexpression significantly reduced CD3^+^CD8^+^ T cell infiltration (p = 0.001, Figure S3, Supporting Information) in pancreatic cancer.

Additionally, further investigation is required to determine whether the antitumor effect of *α*MSLN treatment is dependent on CD3^+^CD8^+^ T cell infiltration. Therefore, in a subcutaneous tumor model established with Panc02 cells, we evaluated the antitumor efficacy of *α*MSLN in combination with a CD8^+^ T cell‐blocking antibody (Figure S4A, Supporting Information). Compared to the IgG isotype control, the CD8‐blocking antibody (*α*CD8) significantly diminished the antitumor efficacy of *α*MSLN (*p* < 0.0001, Figure S4B,C, Supporting Information). Furthermore, ELISA results revealed that the serum level of MSLN was significantly reduced following treatment with *α*MSLN plus IgG compared to PBS treatment (*p* < 0.0001, Figure S4D, Supporting Information). However, co‐treatment with *α*MSLN and *α*CD8 resulted in a slight upregulation of serum MSLN levels (vs *α*MSLN plus IgG, p = 0.0941). These findings suggest that the antitumor activity of *α*MSLN is partially dependent on CD3^+^CD8^+^ T cells, highlighting its potential as a novel strategy to enhance the immunotherapeutic efficacy for pancreatic cancer patients.

### 2.2 Targeting MSLN Enhances Neoantigen Vaccine Induced Tumor‐Specific T Cell Infiltration in Subcutaneous Pancreatic Cancer Model

**Figure 2:**
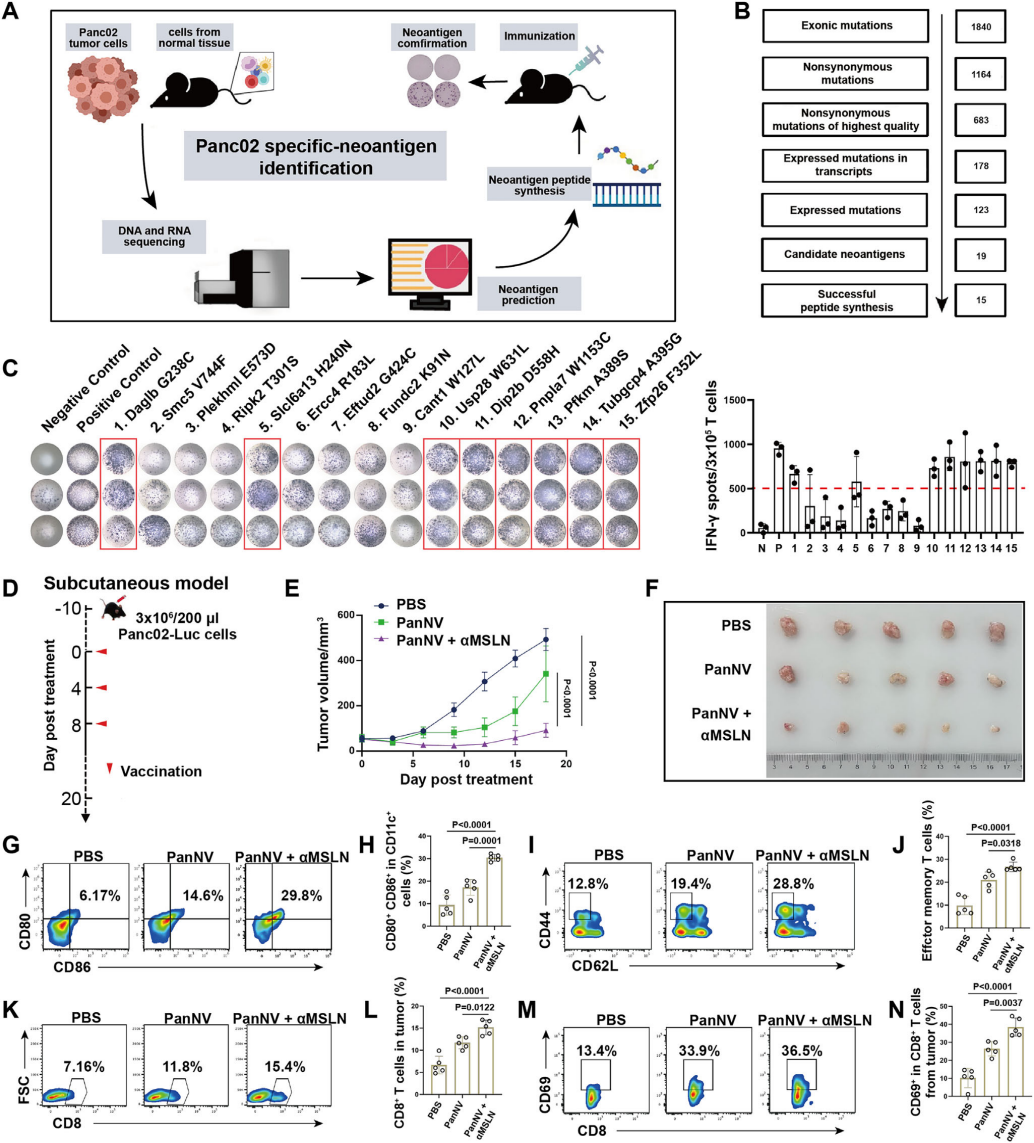
Neoantigen identification and antitumor evaluation in subcutaneous pancreatic cancer. A) Tumor neoepitope identification processes for Panc02 cells. B) Screening workflow of neoantigen peptides. C) Potential immunogenicity of neoantigen peptides identified in Panc02 evaluated by ELISPOT assay. 8 neoantigen peptides with more than 500 spots were selected to develop personalized neoantigen vaccine indicated by dotted line. D) Schematic diagram showing the timeline of subcutaneous pancreatic cancer model construction and treatment. E,F) Tumor volume monitoring in Panc02 tumor‐bearing C57BL/6 mice after receiving PBS, PanNV and PanNV plus *α*MSLN treatment for three times, respectively (n = 5; two‐way ANOVA). G, H) The percentage and statistic analysis of matured DCs with CD80 and CD86 co‐expression in the lymph nodes detected by flow cytometry (n = 5; one‐way ANOVA). I,J) The percentage and statistic analysis of effector memory T cells in splenic CD8^+^ T cells detected by flow cytometry (n = 5; one‐way ANOVA). K‐N) The percentage and statistic analysis of CD8^+^CD69^+^ T cells detected in tumor tissue by flow cytometry (n = 5; one‐way ANOVA). Data are presented as the mean ± SEM. *p < 0.05, **p < 0.01, ***p < 0.001, ****p < 0.0001; ns, no significance.

The infiltration degree of tumor‐specific T cells in tumor sites directly affects their anti‐tumor effect. However, most of the T cells infiltrated into pancreatic cancer are bystander T cells that do not have tumor killing function, while only few tumor‐specific T cells are found. Since the neoantigen vaccine can generate tumor‐specific T cells, we further investigated whether *α*MSLN can enhance the infiltration of neoantigen vaccine‐induced tumor‐specific T cells, and thereby improve anti‐tumor effects. First, the mouse pancreatic cancer cell line Panc02 was utilized as a model to identify potential neoantigens for the preparation of neoantigen vaccine. As shown in **Figure** **2**A, Panc02 tumor and C57BL/6 mouse tail tissues were subjected for whole exome sequencing and transcriptome sequencing, and then potential personalized tumor neoantigen mutations were identified by multiple bioinformatic analysis. A total of 1164 high‐quality non‐synonymous DNA mutations were identified through exome sequencing, with 123 confirmed to be expressed in the transcriptome data (variant allele frequency ≥10%, read depth ≥20 and transcripts per million of the corresponding gene ≥1) (Figure 2B). Then, the binding affinity between these potential neoantigens and MHC‐I (H‐2Kb allele) were evaluated by 8 algorithms as indicated in methods, showing that 19 short peptides (8‐11AA) producing by neoantigen mutations were predicted with high binding affinity for MHC‐I (percentile rank across all MHC binding affinity prediction algorithms ≤2%), and considered as potential panc02 specific neoantigens (Table S1, Supporting Information). These potential neoantigen peptides with high binding affinity were all selected for long peptide preparation (17AA, extending on both sides with the mutated amino acid as the center) and 15 of them were successfully synthesized for immunogenicity evaluation. As shown in Figure 2C, ELISPOT results analysis showed that 8 neoantigen peptides (Daglb_G238C, Slcl6a13_H240N, Usp28_W631L, Dip2b_D558H, Pnpla7_W1153C, Pfkm_A389S, Tubgcp4_A395G and Zfp26_F352L) could elicit strong immune responses (IFN‐*γ* spots > 500) in T cells stimulated by each neoantigen‐pulsed autologous matured dendritic cells (DCs). Moreover, the eight peptides were mixed and used to further immunize normal mice. Subsequently, spleen‐derived T cells were isolated and co‐cultured with Panc02 cells to detect IFN‐*γ* secretion levels. The results showed that T cells isolated from the mice immunized with the neoantigen peptides secreted approximately four times more IFN‐*γ* than T cells derived from the unimmunized mice (Figure S5, Supporting Information). These results suggested that these 8 peptides could be identified as Panc02 specific neoantigens and further mixed with optimized clinically available dual immune adjuvants for preparing Panc02 specific neoantigen vaccine (PanNV).

To evaluate whether *α*MSLN can enhance anti‐tumor effects of neoantigen vaccine, subcutaneous Panc02 mouse model was constructed and further randomly divided into 3 groups to receive PBS, PanNV alone and PanNV plus *α*MSLN treatment on days 0, 4, 8, respectively (Figure 2D). As shown in Figure 2E, the tumor burden of mice in PBS treated group showed a rapid increase over 18 days, while the tumor progression in the PanNV treatment group was reduced (although the p value did not reach 0.05), demonstrating a certain anti‐tumor ability of neoantigen vaccine. As expected, the tumor growth of PanNV plus *α*MSLN treated mice showed most obvious tumor suppression when compared with other two treated groups (both *p* < 0.0001, Figure 2E,F). To characterize potential immune responses in mice treated with PanNV plus *α*MSLN, we further performed flow cytometry to evaluate the proportion of matured DCs (CD11c^+^, CD80^+^ and CD86^+^) in lymph nodes (LNs), effector memory CD8^+^ T cells (CD44^+^ and CD62L^−^) in spleen and the infiltration and activation of T cells in tumor tissue obtained on the day 18, respectively. Significantly, the percentages of matured DCs and effector memory CD8^+^ T cells in the mice treated with PanNV plus *α*MSLN were significantly higher than that in PBS treated group and PanNV treated group (p = 0.0001 and p = 0.0318, Figure 2G–J). Meanwhile, the percentage of CD8^+^ T cell infiltration in tumor was also significantly increased after in the mice treated with PanNV plus *α*MSLN when compared with PanNV treatment (p = 0.0122, Figure 2K,L). Afterwards, we further characterized the expression of CD69 (a marker of T cell resident and activation) on the surface of CD8^+^ T cells in pancreatic cancer tissue, which population is considered to be significantly enriched with neoantigen‐specific T cells in our previous studies.^[^ ^11^ ^]^ As shown in Figure 2M,N, the amount of CD69^+^CD8^+^ T cells in tumor tissue was significantly higher in the combination treatment group compared to the PanNV alone group (p = 0.0037). In addition, to further identify the tumor killing efficiency of the CD69^+^CD8^+^ T cells, the Panc02‐based subcutaneous tumor model was reconstructed and treated with PBS, *α*MSLN, PancNV and the combined (*α*MSLN plus PancNV) therapy, respectively. Then the CD69^+^CD8^+^ T cells were isolated from tumors and further co‐cultured with Panc02 cells for 48h. The cell culture supernatants were collected for cytotoxicity assays using LDH detection. As expected, the CD8^+^CD69^+^ T cells from PanNV plus *α*MSLN treated group exhibited nearly eight times of the tumor‐killing efficiency compared to those from the PBS and *α*MSLN treated groups, and nearly two times of the tumor‐killing efficiency compared to the PancNV treated group (*p* < 0.0001, Figure S6, Supporting Information). These results indicate that target MSLN indeed can enhance neoantigen vaccine induced immune response in pancreatic cancer.

### 2.3 Antitumor Effect of Neoantigen Vaccine Plus MSLN Antibody in Orthotopic Pancreatic Cancer

**Figure 3:**
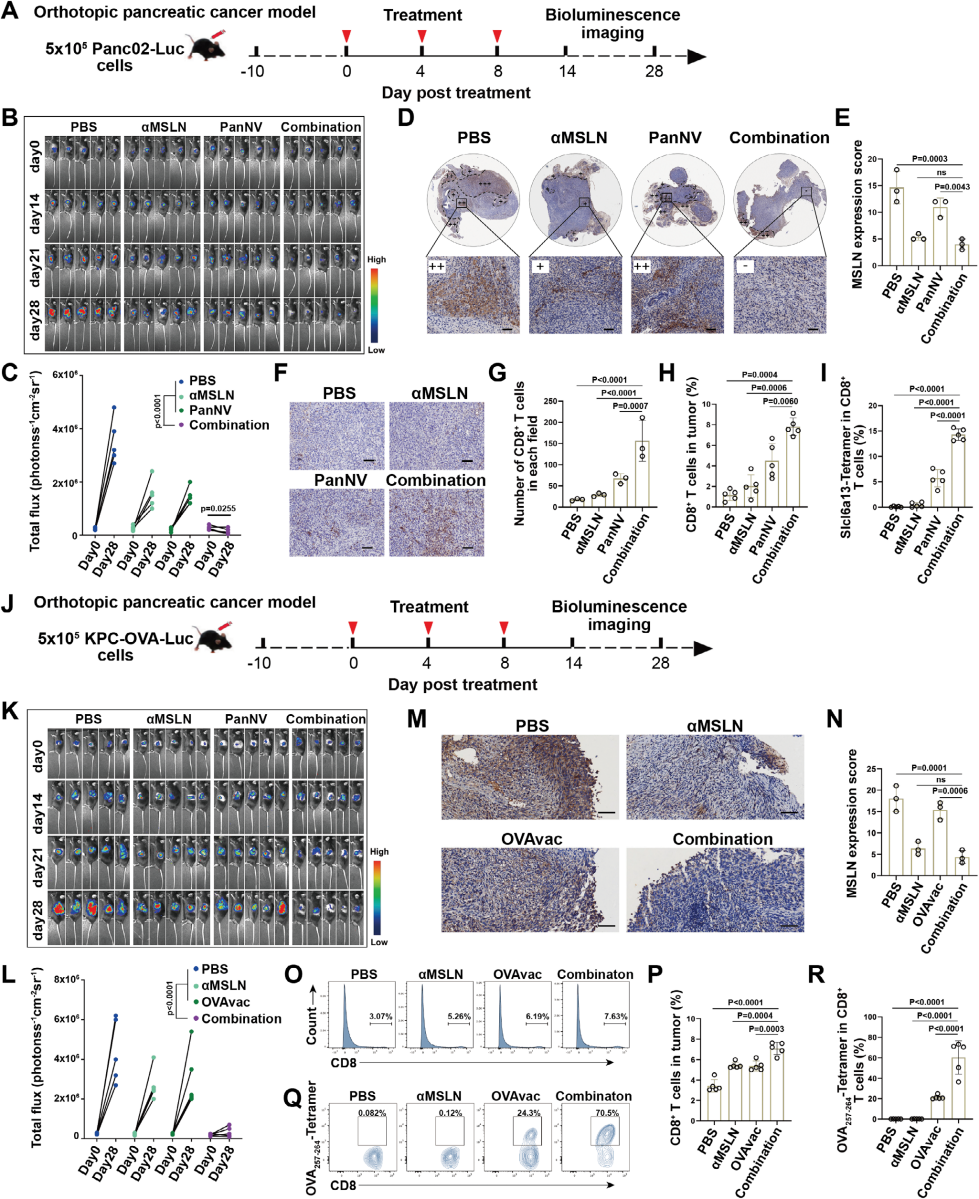
The antitumor effects of the combined treatment of PanNV plus *α*MSLN in orthotopic pancreatic cancer. A) Schematic diagram showing the timeline of orthotopic pancreatic cancer model construction based on Panc02 cells and the treatment procedure. B,C) Tumor burden monitoring and statistic analysis of PBS, *α*MSLN, PanNV, and *α*MSLN plus PanNV (Combination) treated mice by bioluminescence imaging (n = 5; two‐way ANOVA). D,E) The represent graph and statistic analysis of MSLN expression in Panc02 tumor after treatment by IHC staining (n = 3; one‐way ANOVA). Scale bar = 100 µm. F,G) The represent graph and statistic analysis of CD8^+^ T cell infiltration in Panc02 tumor after treatment by IHC staining (n = 3; one‐way ANOVA). Scale bar = 100 µm. H,I) The percentage and statistic analysis of CD8^+^ T cells (H) and Slcl6a13‐specific CD8^+^ T cells (I) in tumor tissues by flow cytometry (n = 3; one‐way ANOVA). J) Schematic diagram showing the timeline of orthotopic pancreatic cancer model construction based on KPC‐OVA‐Luc cells and the treatment procedure. K,L) Tumor burden monitoring and statistic analysis of PBS, *α*MSLN, OVAvac, and *α*MSLN plus OVAvac (Combination) treated mice (n = 5; two‐way ANOVA) by bioluminescence imaging. M,N) The represent graph and statistic analysis of MSLN expression in KPC‐OVA‐Luc tumor after treatment by IHC staining (n = 3; one‐way ANOVA). Scale bar = 100 µm. O‐R) The percentage and statistic analysis of tumor infiltrated CD8^+^ T cells (O, P) and OVA_257‐264_‐specific CD8^+^ T cells (Q, R) in tumor tissues by flow cytometry (n = 5; one‐way ANOVA).

Orthotopic pancreatic cancer possesses a unique microenvironment including numerous fibroblast populations, a dense extracellular matrix, and diverse immunosuppressive immune cell populations. This composition leads to fibrosis and a reduction in blood vessels within the pancreatic tumor, thereby hindering the ability of therapeutic drugs or cells to achieve a sufficient therapeutic concentration within the tumor. Therefore, to better simulate the microenvironment of pancreatic cancer and better evaluate the anti‐tumor effect of PanNV plus *α*MSLN, an orthotopic pancreatic cancer mouse model was constructed by injecting Panc02 into the pancreas, and further treated with PBS, *α*MSLN, panNV, and panNV plus *α*MSLN respectively (**Figure** **3**A). As shown in Figure 3B,C, consistent with the results observed by subcutaneous tumors, the tumors in the MSLN antibody treatment alone or the PanNV treatment alone, although have a certain tumor growth inhibition compared to the PBS treatment, but correspondingly showed a significantly increase of tumor burden compared with that in DAY0. However, the tumor growth of combined treatment showed the most significant inhibition comparing with other 3 treatment groups (all *p* < 0.0001), and the tumor burden also appeared a significant regression and the majority of mice showed partial response (PR) after 28 days comparing with that in DAY0 (p = 0.0255, Figure 3C). This result demonstrated that *α*MSLN plus PanNV could significantly enhance therapeutic effect in the orthotopic pancreatic cancer. To further clarify the role of combined treatment, the expression of MSLN and the infiltration of CD8^+^ T cells in the tumor tissue were analyzed by IHC. Significantly, the expression of MSLN in tumor tissue from mice treated with *α*MSLN plus PanNV was significantly suppressed (similar to the result of MSLN antibody treatment alone) when comparing with the PanNV or PBS treated group (Combine vs PanNV: p = 0.0043, Combine vs PBS: p = 0.0003, Figure 3D,E), and the infiltration level of CD8^+^ T cells was also significantly higher than that in the other three groups (all *p* < 0.001, Figure 3F,G). The enhanced infiltration levels of CD8^+^ T cells in combined treatment were further confirmed by flow cytometry (Figure 3H). To further directly characterize the proportion of potential neoantigen specific CD8^+^ T cells in the pancreatic cancer, a fluorescently labeled tetramer for immunogenic neoantigen peptide Slc16a13 (p. 234–241: YVHLVANL): H‐2Kb (Slc16a13) was applied to detect the infiltrated CD8^+^ T cells that expressed Slc16a13‐specific T‐cell receptors (TCRs). As shown in Figure 3I, the peptide‐MHC tetramer staining revealed that the intratumoral Slc16a13‐specific CD8^+^ T cells in mice received combined therapy were much higher than that in other three groups (all *p* < 0.0001). Overall, these results indicate that targeting MSLN can significantly enhance the infiltration of induced neoantigen specific T cells, thereby enhancing its anti‐tumor efficacy in pancreatic cancer.

Moreover, to further verify the antitumor effect of the combined treatment of MSLN antibody plus neoantigen vaccine, another orthotopic pancreatic cancer model were constructed by KPC‐OVA‐Luc cells. The orthotopic KPC‐OVA‐Luc tumor‐bearing mice were divided into four groups and received PBS, *α*MSLN alone, OVAvac (OVA_250‐264_: SGLEQLESIINFEKL) alone and combined (*α*MSLN plus OVAvac) treatment on days 0, 4, 8, respectively (Figure 3J). As expected, the tumor growth of mice with combined treatment showed most dramatic tumor suppression when comparing with other three treated groups (Figure 3K,L). Significantly, immunohistochemistry for MSLN antibody showed that the expression of MSLN in tumor tissues from mice treated with *α*MSLN combined with OVAvac was significantly suppressed, similar to the group treated with *α*MSLN alone, when compared to the OVAvac or PBS‐treated groups (Combine vs OVAvac: p = 0.0006, Combine vs PBS: p = 0.0001, Figure 3M,N). Moreover, the infiltration level of CD8^+^ T cells was also significantly higher in the combined therapy group compared to other three groups, as confirmed by flow cytometry (all *p* < 0.001, Figure 3O,P). To further characterize the proportion of OVA‐specific CD8^+^ T cells in orthotopic KPC‐OVA‐Luc tumor tissues, a fluorescently labeled tetramer for the immunogenic neoantigen peptide OVA_257‐264_ (SIINFEKL): H‐2Kb was used to detect infiltrating CD8^+^ T cells expressing OVA_257‐264_‐specific T‐cell receptors (TCRs). As shown in Figure 3I, peptide‐MHC tetramer staining revealed that the proportion of intratumoral OVA_257‐264_‐specific CD8^+^ T cells in mice receiving the combined therapy was significantly higher than in the other three groups (all *p* < 0.0001). These findings further suggest that the therapeutic efficacy against pancreatic cancer can be significantly enhanced by combining MSLN antibodies with Neoantigen vaccine. Additionally, to evaluate the clinical translational potential of this combination strategy, the changes of blood biochemical parameters and major organ (heart, liver, spleen, lung, kidney) injuries were studied in mice receiving different treatments as indicated. As shown in Figure S7 (Supporting Information), blood biochemical assays and H&E staining of vital organs showed there was no significant difference between the combined treatment group and the control group (tumor‐free mice) in either blood biochemical indicators or major organ damage changes. These results suggest that *α*MSLN combined with personalized neoantigen vaccine is a potential clinical safe and feasible strategy for pancreatic cancer treatment.

### 2.4 The Dynamic Changes of Immune Microenvironment in Pancreatic Cancer During the Combined Treatment

**Figure 4:**
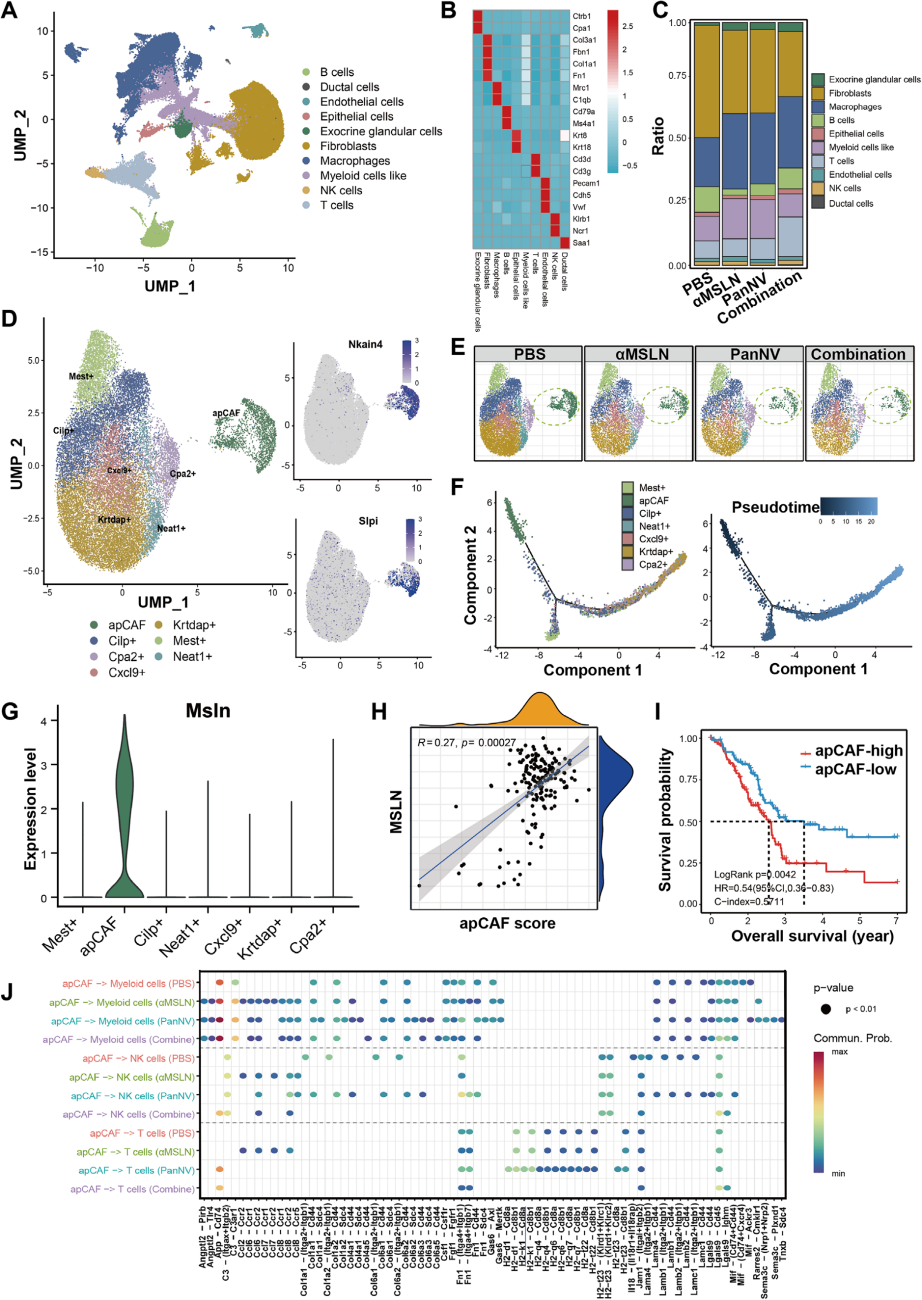
Single‐cell RNAseq revealed alterations of pancreatic tumor microenvironment upon different treatments. A) UMAP plot showing all the identified cell types within the pancreatic tumor. B) Heatmap illustrating the expression levels of marker genes specific to each cell type. C) Proportion of each cell type present in tumors across all four treatment groups. D) UMAP plot showing 7 fibroblast subtypes after re‐clustering. E) UMAP plots of fibroblast cell subtypes separated by treatment condition. F) Pseudotime trajectory of fibroblast subtypes, colored by fibroblast subtypes (left); colored by pseudotime (right). G) Expression level of Msln in each fibroblast subtype. H) The correlation between apCAF score and expression level of MSLN in TCGA PADC cohort. I) Kaplan‐Meier curve of 5‐year overall survival for patients with pancreatic cancer stratified by median expression of apCAF score. J) The interactions between apCAFs and myeloid cells, as well as T cells and NK cells, as suggested by Cellchat.

To further gain insight into the immune microenvironment change of orthotopic pancreatic cancer models after the treatment of *α*MSLN plus neoantigen vaccine, Panc02 tumor tissues from the PBS, *α*MSLN alone, PanNV alone, and PanNV plus *α*MSLN groups were subjected to single‐cell RNA‐seq after 12‐day treatment period. Following the exclusion of low‐quality cells, a total of 71258 cells were retained, with the combined treatment group comprising 17349 cells, the neoantigen vaccine treatment alone group comprising 15399 cells, the *α*MSLN treatment alone group comprising 16719 cells, and the PBS control group comprising 21791 cells. We identified ten cell types including endothelial cells (Pecam1, Cdh5, Vwf), Fibroblasts (Col3a1, Col1a1, Fn1, Acta2, Fbn1), T cells (CD3D, CD3G), B cells (Cd79a, Ms4a1), ductal cells (Saa1), macrophages (Mrc1, C1qb), Epithelial cells (Krt8, Krt18) and a myeloid‐like cell cluster with moderate expression levels of both myeloid and fibroblast cell markers despite lacking apparent canonical markers (**Figure** **4**A,B). Compared with PBS, *α*MSLN alone, and PanNV alone, T cells were enriched while fibroblasts were reduced in tumor tissue collected from the combined treatment group, indicating significant immune activation and growth inhibition of fibroblasts following combined treatment (Figure 4C).

Subsequently, we first extracted and re‐clustered fibroblasts to gain a deeper understanding of the heterogeneity within the fibroblast compartment that associated with the efficacy of immunotherapy. We identified seven clusters of fibroblasts and annotated them based on the dominant cluster‐specific gene apCAFs (Antigen‐presenting Cancer‐Associated Fibroblasts, characterized by specific expression of Slpi and Nkain4), Clip^+^, Cpa2^+^, Cxcl9^+^, Krtdap^+^, Mest^+^ and Neat1^+^ fibroblasts (Figure 4D).^[^ ^12^ ^]^ apCAFs, recognized as a distinct tumor‐associated fibroblast subset that express MHC class II molecules which can directly present antigens to CD4^+^ T cells, have recently been revealed to contribute to immune suppression through the transition of naive CD4^+^ T cells into regulatory T cells (Tregs).^[^ ^13^ ^]^ Notably, apCAFs and Krtdap^+^ fibroblasts exhibited a consistent decrease across all treatment groups, with a particularly pronounced reduction observed in the combined treatment group (Figure 4E; Figure S8, Supporting Information). We further investigated the temporal dynamics of these fibroblast subtypes utilizing the Monocle2 algorithm.^[^ ^14^ ^]^ Pseudotime analysis indicated that apCAFs initiated the trajectory, branching into two distinct evolutionary paths: one was ended at Mest^+^ fibroblasts, and the other culminating at Krtdap^+^ fibroblasts (Figure 4F). The results suggested that the combined treatment could reduce the amount of apCAFs in pancreatic cancer by promoting the transformation of apCAFs into Mest^+^ fibroblasts, and potentially enhance the efficacy of immunotherapy. Furthermore, apCAFs exhibited significantly higher expression of the MSLN gene compared to other fibroblast subtypes, a cell‐surface protein specially targeted by *α*MSLN (Figure 4G). To further confirm whether apCAFs expressed MSLN at high levels, the apCAFs in the tumor samples from Panc02 orthotopic pancreatic cancer mice were sorted using the markers Podoplanin and CD74 (Figure S9A, Supporting Information). qPCR analysis confirmed that the expression level of MSLN in apCAFs was more than five times higher comparing to the unsorted tumor cells (Figure S9B, Supporting Information). Based on the high expression of MSLN in apCAFs, we hypothesized that *α*MSLN treatment could eliminate apCAFs within the tumor. Therefore, we employed multiplex immunofluorescence to label apCAFs and MSLN in Panc02 orthotopic pancreatic tumor samples, and further analyzed the changes in apCAFs following *α*MSLN treatment (Figure S9C, Supporting Information). The results showed that after *α*MSLN treatment, both the number of apCAFs and the expression of MSLN were decreased within the tumor. To verify whether *α*MSLN inhibits apCAFs through antibody‐dependent cellular cytotoxicity (ADCC), we co‐cultured sorted apCAFs (Podoplanin^+^ and CD74^+^ cells) with effector immune cells derived from mouse spleen and assessed the cytotoxicity of effector cells against apCAFs in the presence of *α*MSLN (Figure S9D, Supporting Information). We found that the levels of LDH in the supernatant of co‐cultures between apCAFs and effector immune cells significantly increased with increasing concentrations of *α*MSLN accordingly. These results indicated that the reduction of apCAFs was primarily due to *α*MSLN‐mediated ADCC.

Consistently, a significant correlation was observed between the expression level of MSLN and the apCAF score, which is calculated using single‐sample Gene Set Enrichment Analysis (ssGSEA) with top20 marker genes derived from apCAF cluster, within TCGA pancreatic cancer samples (p = 0.00027, Figure 4H). These findings further support the notion that apCAFs are the target affected by the combined treatment involving *α*MSLN. Meanwhile, pancreatic cancer patients exhibiting lower expression level of top 20 upregulated genes from apCAF subtype had significantly longer overall survival time (p = 0.0042, Figure 4I). Interestingly, apCAFs from the combined treatment group exhibit less interaction with naive T cells in ligand‐receptors associated with antigen presentation when compared with other groups (Figure 4J). This observation indicated that the combined PanNV and *α*MSLN treatment might function by inhibiting the interaction between apCAFs and naive T cells.

### 2.5 The Relationship Between Tumor‐Infiltrating T Cells and apCAFs

**Figure 5:**
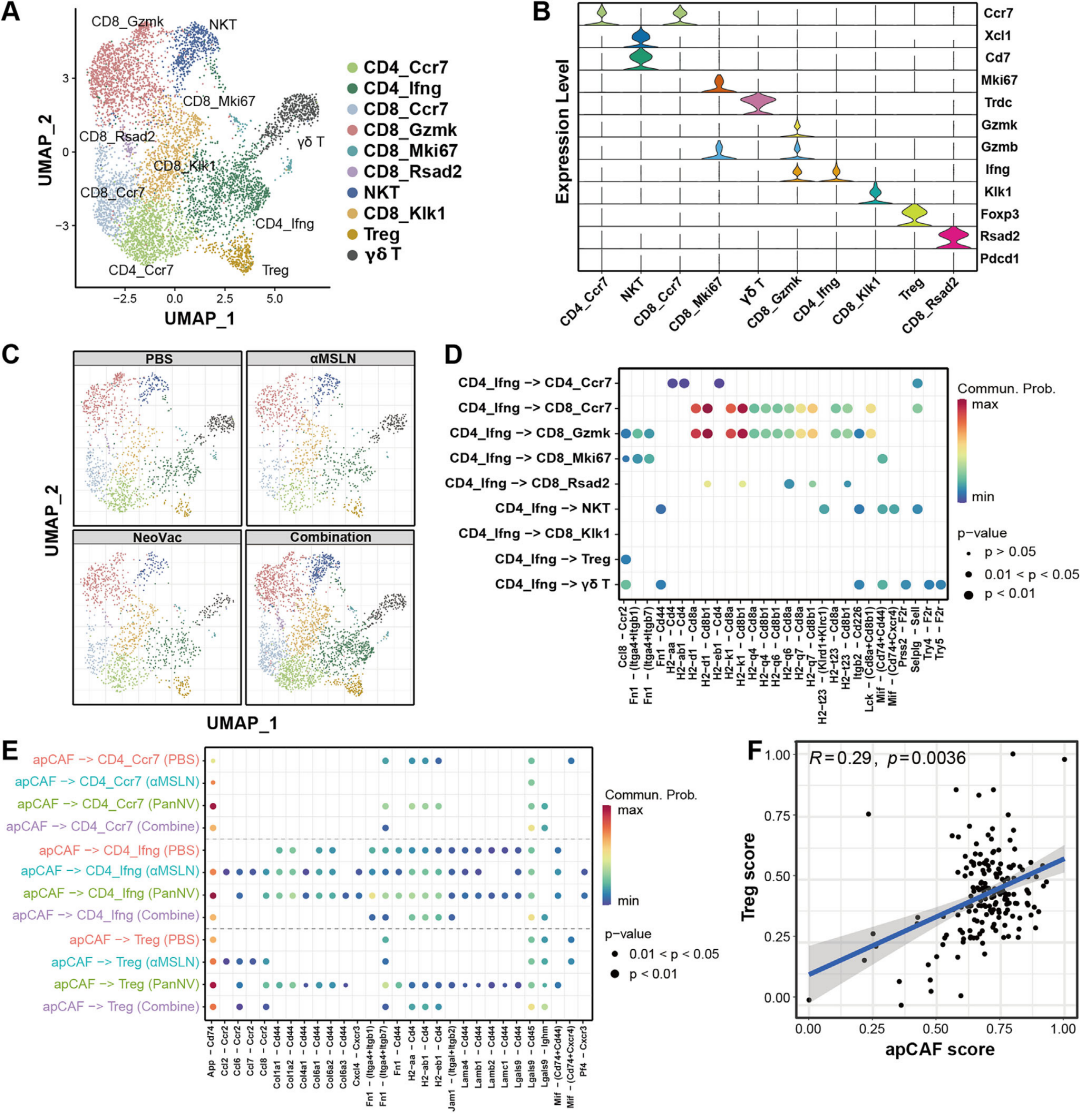
Dynamics of tumor infiltration T cells induced by anti‐tumor treatments. A) UMAP visualization of all the tumor infiltrating T cell subtypes. B) Expression level of marker genes in each T cell subtype. C) UMAP visualizations depicting the distribution of T cell subtypes grouped by treatment methods. D) Cell–cell interactions between CD4_Ifng T cells and other T cell subsets computed using Cellchat. E) Significant ligand‐receptor interactions between apCAFs and all the CD4^+^ T cell subsets as indicated by Cellchat. F) The correlation between apCAF score and Treg score in the TCGA PADC cohort.

Observing a comprehensive upregulation (Figure 4C) and a distinctive interaction pattern with apCAFs in the combined treatment, we subsequently reclustered T cells to gain a more profound understanding of their features. Based on the expression of marker genes, we identified 5 clusters of CD8^+^ T cells (CD8_Ccr7, CD8_GzmK, CD8_Mki67, CD8_Rasd2, CD8_Klk1), 3 three clusters of CD4^+^ T cells (CD4_Ccr7, CD4_Ifng and Treg), NKT and *γ*δT cells (**Figure** **5**A,B). CD8_Gzmk T cells, characterized by elevated expression levels of cytotoxic markers (Gzmb and Ifng) and without expression of dysfunction maker (Pdcd1), are potential neoantigen vaccine‐induced T cells. Its number was obviously upregulated in the combined treatment group, which is consistent with the result in Figure 3E‐G. Interestingly, CD4_Ifng T cells were observed with an obvious upregulation in the combined treatment group, which may be also associated with the enhanced efficacy (Figure 5C; Figure S10, Supporting Information). Analysis of cell–cell interactions analysis revealed a pronounced association with CD8_Gzmk T cells and CD8_CCR7 T cells in ligand‐receptors associated with antigen presentation (CD8 molecule and MHC alleles, Figure 5D). The interaction between CD8_Gzmk T cells and CD4_Ifng T cells suggests a potential T cell helper role of CD4_Ifng T cells in augmenting the cytotoxic activity of CD8_Gzmk T cells.

Previous study have demonstrated that apCAFs could directly bind to and stimulate naive CD4^+^ T cells to differentiate into regulatory T cells.^[^ ^9^ ^]^ Pseudotime analysis indicated that CD4_CCR7 T cells (naive CD4^+^ T cells) initiated the trajectory, branching into two distinct evolutionary paths: one was ended at Treg cells, and the other culminating at CD4_Ifng T cells (Figure S11, Supporting Information). To explore the interaction between apCAFs and CD4^+^ T cells, cell–cell interactions were conducted to analysis involving of apCAFs and all subsets of CD4^+^ T cells. As shown in Figure 5E, the apCAFs derived from the combined therapy and *α*MSLN alone exhibited reduced interaction with CCR7_CD4^+^ T cells when compared with the PBS and PanNV group. The interaction mediated by ligand‐receptors associated with antigen presentation (CD4 molecule and MHC alleles) was entirely absent in both the combined treatment group and the *α*MSLN monotherapy group. These results indicated that the inhibition of apCAFs by *α*MSLN could led to decreased interaction with naive T cells. Consistently, a positive association between the apCAFs score and Treg score was observed in the TCGA pancreatic cancer cohort (p = 0.0036, Figure 5F), which was further validated in another pancreatic tumor cohort (p = 0.00027, Figure S12, Supporting Information).^[^ ^15^ ^]^ Taken together, our results suggested that the anti‐MSLN antibody could reduce the amount of apCAFs in pancreatic cancer and convert Naive T Cells to Treg cells, thereby enhancing the infiltration and anti‐tumor ability of neoantigen vaccine induced CD8^+^ T cells.

### 2.6 Negative Correlation Among apCAFs, CD8^+^ T Cells and IFN‐*γ* ^+^CD4^+^ T Cells in Human Pancreatic Cancer

**Figure 6:**
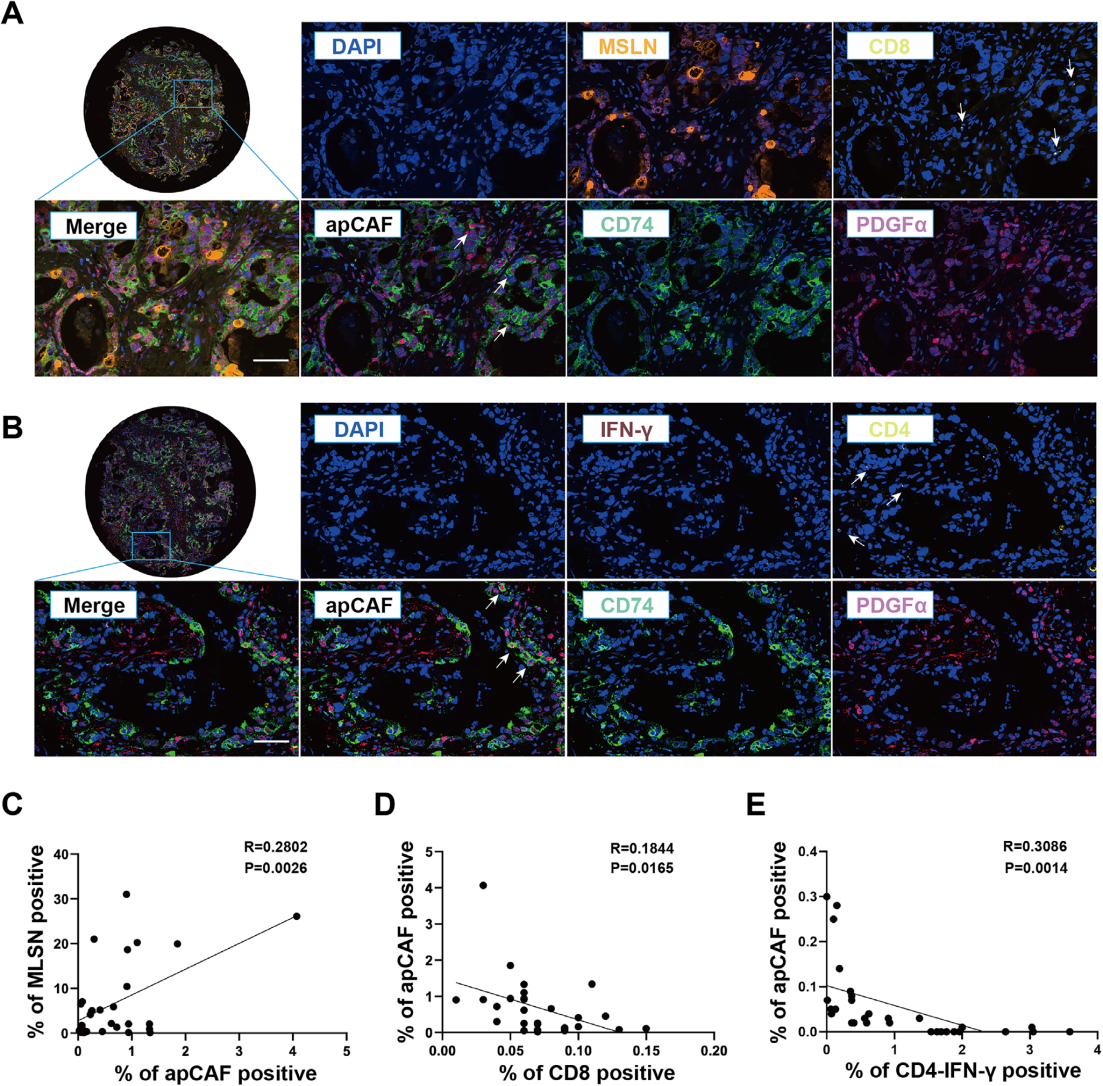
Negative correlation among apCAFs, CD8^+^ T cells and IFN‐*γ* ^+^CD4^+^ T cells in pancreatic cancer. A) The represent graph of the expression of MSLN, CD8, CD74, and PDGF*α* by multiple immune fluorescent staining in tissue microarray, respectively. Scale bar = 100 µm. B) The represent graph of the expression of IFN‐*γ*, CD4, CD74, and PDGF*α* by multiple immune fluorescent staining of tissue microarray. Scale bar = 100 µm. C) The correlation analysis between MSLN expression and apCAF. D) The correlation analysis between apCAFs and CD8^+^ T cells. E) The correlation analysis between apCAFs and IFN‐*γ* ^+^CD4^+^ T cells.

The aforementioned animal results suggest that targeting apCAFs by *α*MSLN can reverse the immunosuppressive microenvironment of pancreatic cancer, whereas the correlation among apCAFs, CD8^+^ T cells and IFN‐*γ* ^+^CD4^+^ T cells in pancreatic cancer remain unclear. To clarify the correlation among apCAFs, CD8^+^ T cells and IFN‐*γ* ^+^CD4^+^ T cells in human pancreatic cancer, multiple immune fluorescent staining with MSLN, CD8, CD74 plus PDGF*α* antibodies and CD4, IFN‐*γ*, CD74 plus PDGF*α* antibodies were performed in 30 cases of pancreatic cancer TMA, respectively. As shown in **Figure** **6**, the amount of apCAF (CD74^+^, PDGF*α* ^+^) in pancreatic cancer was positively correlated with MSLN expression (P = 0.0026), but were negatively correlated with the amount of effector immune cells such as CD8^+^ T cells (p = 0.0165) and IFN‐*γ* ^+^CD4^+^ T cells (p = 0.0014). Combining the antitumor efficiency of anti‐MSLN antibody plus neoantigen vaccine in two orthotopic pancreatic cancer model and the results of multiple immunofluorescences in human pancreatic cancer, showed that MSLN^+^ apCAFs in pancreatic cancer could significantly inhibit the infiltration of effector immune cells and correspondingly the combined treatment of *α*MSLN plus personalized neoantigen vaccine might serve as a feasible and effective strategy to improve immunotherapy of pancreatic cancer.

## 3 Discussion

MSLN is highly expressed in most pancreatic cancer cells and is considered to be an attractive therapeutic target. Presently, most studies focus on the effects of targeting MSLN on pancreatic cancer cells themselves, while whether it can improve the infiltration of immune cells in pancreatic cancer is rarely studied.^[^ ^6^, ^8^, ^16^ ^]^ In this study, we first confirmed that the expression of MSLN in pancreatic cancer showed significantly negative correlation with the infiltration of cytotoxic T cells through three datasets and multicolor immunofluorescence of TMA, and demonstrated in vivo that killing the cells with MSLN expressing could improve the infiltration of T cells in a certain extent, suggesting that targeting MSLN could kill tumor cells expressing MSLN and modulate the immune environment of pancreatic cancer. Because pancreatic cancer itself is an immune desert, there is an extreme lack of tumor specific effector T cell infiltration.^[^ ^17^ ^]^ Therefore, we established a novel combination immunotherapy strategy here, that is, antibodies targeting MSLN combined with personalized neoantigen vaccines. Tumor neoantigen vaccine is firstly used to induce tumor‐specific T cell generation in vivo, and then combined with MSLN‐targeting antibodies to break the immune tolerance microenvironment of pancreatic cancer, so as to enhance the infiltration of tumor‐specific T cells in pancreatic cancer to achieve the purpose of synergism.

The special immunosuppressive microenvironment of pancreatic cancer includes the encapsulation of a large number of CAFs, myeloid cells, lymphocytes, and vascular endothelial cells, which limits the efficacy of immunotherapy in pancreatic cancer.^[^ ^18^ ^]^ CAFs are critical drivers of pancreatic cancer progression.^[^ ^19^ ^]^ Some CAFs subsets can directly inactivate the immune system by expressing PD‐L1 or secreting prostaglandin E2, which could impair the tumor‐killing effect of NK cells or T cells.^[^ ^19^, ^20^ ^]^ Moreover, CAFs also could communicate with other stromal cells and tumor cells through the secretion of various cytokines, inhibiting immune cell function and promoting tumor development.^[^ ^19b^ ^]^ Finally, they can also regulate the extracellular matrix, creating a barrier for the infiltration of drugs and immune cells.^[^ ^21^ ^]^ Huocong Huang et al. reported the presence of an apCAF subtype that expresses high levels of MSLN in pancreatic cancer.^[^ ^9^ ^]^ These apCAFs originate from mesothelial cells, which typically form protective membranes around organs, body cavities, and tissues. During the development of pancreatic cancer, mesothelial cells proliferate and acquire fibroblastic characteristics, further transforming into apCAFs.^[^ ^9^ ^]^ Moreover, trauma‐ or inflammation‐related signals, such as IL‐1 and TGF*β* signaling pathways, have been found to play crucial roles in driving the transformation of mesothelial cells into apCAFs.^[^ ^22^ ^]^ Furthermore, apCAFs are capable of expressing MHC‐II molecules and presenting antigens to CD4^+^ T cells. However, they lack classical costimulatory molecules such as CD40, CD80, and CD86, which are necessary for the full activation and clonal expansion of CD4^+^ T cells following T cell receptor (TCR) engagement.^[^ ^9^ ^]^ Therefore, apCAFs are able to induce naive CD4^+^ T cells to enter the differentiation pathway of Tregs, further inhibiting the activity of other immune cells, including CD8^+^ T cells for facilitating tumor evasion of the immune system. Due to the significant role of apCAFs in the tumor microenvironment, targeting apCAFs to lift the immunosuppressive state of the pancreatic cancer microenvironment can help improve the treatment outcomes of pancreatic cancer. In this study, we also found that apCAFs were negatively correlated with cytotoxic T cells and IFN‐*γ* ^+^CD4^+^ T cells in pancreatic cancer tissues. This suggests that targeting apCAFs can provide novel therapeutic strategies in pancreatic cancer treatment. Through single‐cell sequencing, we discovered that antibodies targeting MSLN effectively inhibit the generation of apCAFs and block the conversion of naive CD4^+^ T cells to Tregs, thereby enhancing the antitumor activity of T cells induced by neoantigen vaccines.

In conclusion, we found that targeting MSLN could improve the degree of T cell infiltration. Moreover, combining anti‐MSLN antibody with personalized neoantigen vaccine could significantly enhance the immunotherapy efficacy of pancreatic cancer by reducing apCAF cells to interrupt the conversion of naive CD4^+^ T cells to Tregs, and therefore increasing the infiltration of tumor‐specific T cells. Taken together, our therapeutic strategy of combining MSLN‐targeted antibody with personalized neoantigen vaccine could provide a new perspective for improve therapeutic outcomes by efficiently relieving the immunosuppressive state of pancreatic cancer microenvironment.

## 4 Experimental Section

### Correlation Analysis Between MSLN Expression and CD8^+^ T Cell Infiltration in Pancreatic Cancer

The bulk RNA‐seq and clinical data of TCGA pancreatic adenocarcinoma (PAAD) cohort were download from UCSC xena (https://xena.ucsc.edu/).^[^ ^23^ ^]^ 178 pancreatic adenocarcinoma patients with both available transcriptome data and clinical information were included. Additionally, two other expression datasets (GSE28735^[^ ^24^ ^]^ and GSE62452^[^ ^25^ ^]^) of pancreatic cancer were retrieved from GEO database, followed by extraction of the tumor samples (n = 45 and n = 69, respectively). Spearman's rank correlation was employed to assess the correlations between different genes and/or signatures. CD8^+^ T infiltration in bulk RNA seq datasets was evaluated through single‐sample Gene Set Enrichment Analysis (ssGSEA), using gene signature consisted of CD8A and CD8B. The expression level of the CD4 gene was used to represent CD4^+^ T cell infiltration. For survival analysis, patients were stratified into two groups based on the median expression level of the apCAF score. Survival curves between the two groups were estimated using the Kaplan‐Meier method, and the significance was determined using the log‐rank test.

### Cell Lines

Panc02 cells were obtained from ATCC and identified by Zhong Qiao Xin Zhou Biotechnology Co., Ltd (Shanghai, China) with STR reports. KPC‐OVA‐Luc cell line (Pdx1‐cre/LSL‐KrasG12D/P53R172H with overexpression of chicken ovalbumin antigen and luciferase, Cat. NO. NMC‐230049) was purchased from Shanghai Model Organisms Center, Inc. All these cells were cultured in DMEM containing 10% FBS and 1% penicillin/streptomycin at 37 °C in a humidified environment with 5% CO_2_. Luciferase or MSLN‐expressing Panc02 (Panc02‐Luc or Panc02^MSLN+^) cells were established through lentivirus transfection expressing luciferase reporter gene or mouse MSLN, respectively, and then the positive cells were selected by puromycin and maintained in low concentration of puromycin (Shanghai Genechem Co., Ltd).

### Neoantigen Identification

DNA and RNA were extracted from the Panc02‐Luc cell line and C57BL/6 mouse tail tissue using Genomic DNA kit (Tiangen biotech, China) and EasyPure RNA kit (Transgen Biotech, China) according to the manufacturer's instructions, and further subjected to perform whole exome sequencing and transcriptomic sequencing to identify potential personalized neoantigens for Panc02 using SureSelect XT Mouse All Exon Kit and RNA samples were subjected to RNA library preparation using VAHTS Stranded mRNA‐seq Library Prep Kit, respectively. The subsequent sequencing stage was executed on the Illumina Novaseq 6000 platform by Fulgent Co., Ltd, employing paired‐end reads of 150 base pairs.

For exome sequencing data, it performed alignment to the mouse genome (mm10) using the BWA algorithm.^[^ ^26^ ^]^ Following that, duplicate reads were detected and eliminated using Picard in Genome Analysis Toolkit (GATK).^[^ ^27^ ^]^ To identify somatic mutations specific to panc02 cell line, it employed Mutect2 and utilized the C57BL/6 mouse tail tissue as the normal reference. Somatic mutations were further annotated using Variant Effect Predictor (VEP), and missense mutations were filtered and selected for neoantigen prediction using the pVAC‐Seq pipeline.^[^ ^28^ ^]^

For each missense mutation, the binding affinity of 8–11 mer peptide containing mutated amino acids to H2Kb allele was predicted using a comprehensive set of algorithms, including NetMHCpan, NetMHC, NetMHCcons, PickPocket, MHCflurry, SMM, SMMPBMC and MHCnuggetsI. Transcriptome sequencing data was aligned to the mouse genome (mm10) using the STAR aligner, along with the GENCODE gene annotation.^[^ ^29^ ^]^ Expression levels of all genes were then quantified using the RSEM algorithm, which calculates the metric of transcripts per million (TPM).^[^ ^30^ ^]^ Finally, combing information from both DNA and RNA sequencing data, mutations fulfilling the following criteria were considered as candidate neoantigens: 1) mutations with median binding affinity percentile rank across all MHC binding affinity prediction algorithms ≤2%; 2) mutations with ≥ 20 × depth and variant allele frequency (VAF) ≥ 0.1 in LLC tumor cells at both DNA and RNA level; 3) mutations with ≥ 20 × depth and variant allele frequency (VAF) ≤ 0.01 in normal reference; 4) mutations located at genes and transcripts with TPM ≥1; 5) mutations with DNA frequency ≤0.6 (in order to exclude possible germline mutations).

### Enzyme‐Linked Immunospot (ELISPOT) Assay

Mouse splenic T cells' IFN‐*γ* secretion was detected by ELISPOT kit (Mabtech, 3321–4APT‐10), similar to the previous reports.^[^ ^11^, ^31^ ^]^ Briefly, at day 0, 2 million mouse bone marrow derived monocytes/well were added into a 6‐well plate and cultured with 2 mL RPMI‐1640 medium/well (10 ng mL^−1^ IL‐4 and 20 ng mL^−1^ GM‐CSF, R&D systems) to induce bone marrow derived‐DCs (BMDCs) at 37 °C with 5% CO_2_. Half of the medium volume was changed at day 3. At day 6, BMDCs were divided to pulse with single Panc02 cell‐specific neoantigen peptide (4 µg per peptide) for 48 h. For ELISPOT assay, 3 × 10^4^ BMDCs disposed by single neoantigen peptide were co‐incubated with 3 × 10^5^ splenic T cells in a multiscreen 96‐well filtration plate (Mabtech, 3321‐4APT‐2) at 37 °C with 5% CO_2_ for another 48 h. The splenic T cells used here were isolated from the mice immunized three times by the Panc02 cell‐derived neoantigen peptide pools (Pool 1: Daglb, Smc5, Plekhml, Ripk2, Slcl6a13; Pool 2: Ercc4, Eftud2, Fundc2, Cant1, Usp28; Pool 3: Dip2b, Pnpla7, Pfkm, Tubgcp4, Zfp26, 100 µg per peptide). BMDCs pulsed with PBS served as a negative control, while splenic T cells co‐incubated with CD3 antibody for T cell activation served as a positive control. The plates were washed and subsequently incubated with detection antibody (R4‐6A2‐biotin, 1 µg mL^−1^, 100 µl/well) for 2 h at room temperature and the subsequent operations were carried out according to the instructions. Finally, IFN‐*γ* spot‐forming cells were imaged and analyzed by ELISPOT Analysis System (AT‐Spot‐2200, Beijing Antai Yongxin Medical Technology Co., Ltd). A score of IFN‐*γ* spot‐forming cells above 500 was considered positive.

For the co‐culture of T cells and Panc02 tumor cells, it first performed pre‐immunization of C57BL/6 mice with the neoantigen peptide vaccine as described above, followed by isolation of spleen‐derived T cells for later use. The T cells isolated form the unimmunized mice were use as negative control. Panc02 cells (1 × 10^4^ per well) were first plated; after 24 h, the spleen‐derived T cells (1 × 10^5^ per well) were co‐cultured with Panc02 cells. After 48 h, the cells were digested with trypsin and removed from the wells, followed by IFN‐*γ* detection and analysis according to the aforementioned method.

### In Vivo Tumor Models and Evaluation of Antitumor Efficacy

The tumor challenges were performed by injecting Panc02‐Luc cells or KPC‐OVA‐Luc into 6–8 weeks old female C57BL/6 mice purchased from Shanghai SLAC Laboratory Animal (License number: SCXK(Hu) 2022‐0004, Shanghai, China). The subcutaneous pancreas cancer model was established by subcutaneous injection of 3 × 10^6^ Panc02‐Luc cells and KPC‐OVA‐Luc into the right axilla. An orthotopic pancreas cancer model was established by direct intrapancreatic injection of 5 × 10^5^ Panc02‐Luc cells or KPC‐OVA‐Luc cells. Tumor monitoring in the subcutaneous pancreas cancer model was performed by a single operator every 5 days in two dimensions using calipers. Tumor volume was calculated from the measured data as follows: V = AB^2^ /2 (A is the long diameter and B is the short diameter). When the tumor volume grew to 50–80 mm^3^, recorded as day 0, the mice were randomly divided into 3 groups (n = 5) to initiate treatment. The experimental group received a neoantigen peptide vaccine comprising eight identified neoantigen peptides (100 µg/peptide/mouse), along with the optimized clinically available dual immune adjuvants (Poly(I:C) (50 µg/mouse) plus R848 (50 µg/mouse)), *α*MSLN (50 µg/mouse, clone number: B35, LSBIO, USA), and *α*CD8 (50 µg/mouse, Cat# C380, Leinco Technologies, Inc.). The tumor bearing mice were given subcutaneous administration (neoantigen vaccines) or tail vein injection (*α*MSLN/*α*CD8) starting on day 0, once every four days for three times. Control groups were treated for the same duration, and the injected drugs were phosphate buffered saline (PBS).

For the orthotopic pancreas cancer model, mice were randomly divided into 4 groups (n = 5) after 10 days of tumor inoculation (recorded as day 0) and then the treatment was initiated. The vaccines of PanNV or OVAvac and *α*MSLN were administered in the same manner as the subcutaneous pancreas cancer experimental group. For PanNV, the information for the peptide sequence is listed in Table S1 (Supporting Information). The peptide sequence for OVAvac was peptide_250‐264_: SGLEQLESIINFEKL. At the end of treatment, mice were sacrificed by cervical dislocation and tumor volume was recorded. In addition, lymph nodes, spleens and tumor tissues were isolated and prepared into single cell suspensions for flow cytometry analysis. A portion of the tumor tissue was excised for formalin‐fixed paraffin embedding followed by immunofluorescence and immunohistochemistry.

### Flow Cytometry

Single cell suspensions from lymph nodes, spleens and tumors were stained with different combinations of antibodies for 30 min under dark, including anti‐mouse CD3‐APC (or FITC) mAb (eBioscience, Cat# E‐AB‐F1013E/ Cat# E‐AB‐F1013C), anti‐mouse CD8‐PE (or FITC) mAb (eBioscience, Cat# E‐AB‐F1104D/ Cat# AN00331C), anti‐mouse CD4‐PE mAb (eBioscience, Cat# AN00417D), anti‐mouse CD11c‐APC mAb (eBioscience, Cat# E‐AB‐F0991E), anti‐mouse CD80‐PE mAb (eBioscience, Cat# E‐AB‐F0992D), anti‐mouse CD86‐PE‐Cyanine7 mAb (eBioscience, Cat# E‐AB‐F0994UH), anti‐mouse CD44‐PE‐Cyanine7 mAb (eBioscience, Cat# E‐AB‐F1100H), anti‐mouse CD62L‐PerCP/Cy5.5 mAb (eBioscience, Cat# E‐AB‐F1011J), and anti‐mouse CD69‐APC mAb (eBioscience, Cat# E‐AB‐F1187E). After staining, the samples were washed two times by PBS and finally resuspended by 300 µl PBS for detection. The anti‐mouse Slc16a13 (p. 234–241: YVHLVANL)‐specific tetramer‐PE was purchased from BioReagent Unit of Cancer Research Center of Xiamen University (Xiamen, China). Tumor single cell suspensions were stained with 2 µL tetramer for 20 min at room temperature, then anti‐mouse CD8 antibody was added and incubated for another 20 min at room temperature. All samples were analyzed on a flow cytometer (BD FACSVerseTM, USA) and data were analyzed by FlowJo v.10.

### Flow Cytometric Sorting

Single‐cell suspensions were undergone Fc blocking with PBS containing 5% BSA for 10 min on ice. Then, the samples were further stained with anti‐mouse CD8‐PE (or FITC) mAb (eBioscience, Cat# E‐AB‐F1104D/ Cat# AN00331C), anti‐mouse CD69‐APC mAb (eBioscience, Cat# E‐AB‐F1187E), anti‐mouse Podoplanin‐APC mAb (Biolegend, Cat# 127410) or anti‐mouse CD74 mAb‐Alexa Fluor 488 (Biolegend, Cat# 151006) in the dark for 30 min on ice, and subsequently washed twice with PBS. Sorting was performed on a Fusion cell sorter (BD FACSAria, USA).

### Cytotoxicity Testing of CD8^+^CD69^+^ T Cells Against Tumor Cells

The Panc02‐based subcutaneous tumor model was established by injection of 3 × 10^6^ Panc02 cells as described previously and treated with PBS, *α*MSLN, PancNV and the combined (*α*MSLN plus PancNV) therapy, respectively. Mice were sacrificed and the tumors were extracted after 20 days of initial treatment. The CD8^+^CD69^+^ T cells from different treatment group were sorted using flow cytometry. Then, Panc02 cells (1 × 10^4^ cells per well) were co‐incubated with CD8^+^CD69^+^ cells (3 × 10^4^ cells per well) in 96‐well plates containing IL‐2 for 48 h in a cell incubator. The cell culture supernatant was then collected for LDH detection and cytotoxicity analysis according to the product manual (MK401, Takara). Briefly, 100 µL sample was transferred into a 96‐well flat bottom plate following 100 µL reaction mixture. Afterwards, the plate was incubated at room temperature for 30 min protected from light. Finally, the absorbance of the samples at 490 nm was measured. The cytotoxicity was calculated by the equation: Cytotoxicity = (Absorbance (sample) – Absorbance (low control)) / (Absorbance (high control) – Absorbance (low control)) * 100%.

### Antibody‐Dependent Cellular Cytotoxicity (ADCC) Assay

Mouse spleen‐derived cells used as effector cells were cultured in RPMI‐1640 medium supplemented with 10% serum. The apCAFs after sorting by the markers of Podoplanin and CD74 were cultured in DMEM medium supplemented with 10% serum. Afterwards, the effector cells (2 × 10^5^ cells per well) were co‐incubated with apCAFs (5 × 10^4^ cells per well) in 96‐well plates containing different concentration of *α*MSLN (1, 5, 25, 50, 100 and 200ng mL^−1^) for 24 h. The cell culture supernatant was collected for lactate dehydrogenase (LDH) detection and cytotoxicity analysis according to the product manual (MK401, Takara). Briefly, 100 µL sample was transferred into a 96‐well flat bottom plate following 100 µL reaction mixture. Then, the plate was incubated at room temperature for 30 min protected from light. Finally, the absorbance of the samples at 490 nm was measured. The cytotoxicity was calculated by the equation: Cytotoxicity = (Absorbance (sample) – Absorbance (low control)) / (Absorbance (high control) – Absorbance (low control)) * 100%.

### mRNA Isolation and Quantitative PCR

Total RNA was extracted from tumor samples or apCAFs using TRIzol Reagent (Invitrogen, Carlsbad, CA, USA) according to the manufacturer's instructions. A total of 1 µg of RNA was used as a template for single‐strand cDNA synthesis. Quantitative PCR (qPCR) for MSLN (For: GAAATTTGTGGCCAGATCTT, Rev: TGCAGGGCATCCAGGGTGGA) was performed on an Applied Biosystems 7300 Real‐Time PCR System (Applied Biosystems, Foster City, CA, USA) using SYBR Green (Invitrogen, USA). The PCR program consisted of an initial denaturation at 95 °C for 10 min, followed by 40 cycles of 95 °C for 15 s, 60 °C for 30 s, and 72 °C for 30 s. qPCR data were analyzed using the 2^−ΔΔCt^ method, with GAPDH (For: AACGACCCCTTCATTGAC, Rev: TCCACGACATACTCAGCAC) as an internal control. All reactions were performed in triplicate.

### Immunohistochemistry

Standard immunohistochemical procedures were used. Fixation, embedding in paraffin, sectioning, deparaffinization, antigen repair, inactivation and blocking were performed on mouse pancreatic cancer tissues. The sections were incubated with the following antibodies: Mesothelin (Thermo Fisher Scientific, PA5‐79698, 1:2000), CD8 (Abcam, ab217344, 1:2000) and CD4 (Abcam, ab183685, 1:500). After incubation with the primary antibody overnight at room temperature, the incubation with the appropriate secondary antibodies were performed for 1 h. The sections were dropped with DAB chromogenic agent, nucleated with hematoxylin and sealed with paraffin. The MSLN expression level was blindly scored from 0 (‐) to 3 (+++) as follows: 0, no signs of MSLN; 1, the expression of MSLN exhibits a scattered distribution; 2, the expression of MSLN exhibits a patchy distribution; 3, the entire field was MSLN positive. The final MSLN expression level for each slice was derived from the total score, and the statistical results were based on three separate analyses. The positive CD8^+^ T cells were analyzed by ImageJ software. The index of CD8^+^ T cells was given as the number of positive nuclei per visual field. Three visual fields per sample were randomly selected for analysis.

### Multi‐Color Immunofluorescence

Multi‐color immunofluorescence was performed according to Opal 6‐Plex Detection Kit (Akoya Biosciences, NEL821001KT). Slides were stained with Mesothelin (Abcam, ab196235, 1:400), CD8 alpha (cab217344, 1:100), CD4 (Abcam, ab183685, 1:100), CD74 (Abcam, ab9514, 1:100), interferon (IFN)‐*γ* (GeneTex, GTX66714, 1:200) and PDGF Receptor *α* (Cell Signaling Technology, 3174, 1:100). Cell nuclei were counterstained with DAPI. Images of immune‐stained slides were collected and analyzed by PhenoCycler‐Fusion 2.0 (Akoya Bioscience). The phenoptrReports R Package was downloaded from the official Akoya website to analyze the obtained data.

### Single‐Cell RNA Library Preparation and Sequencing

Fresh tumor tissues were collected from orthotopic pancreatic cancer mouse models subjected to 14‐day treatment with neoantigen vaccine and/or *α*MSLN, along with a PBS‐treated control group. Upon collection, the tissues were promptly chilled on ice and digested using 2 mL of sCelLiveTM Tissue Dissociation Solution (Singleron, China). The resulting cells were suspended in phosphate‐buffered saline (PBS, HyClone, Marlborough, MA, USA). Cell viability was assessed using the Trypan blue exclusion test (Gibco, Grand Island, NY, USA), with samples demonstrating viability above 80% deemed suitable for subsequent experiments. The cell suspension was further diluted in PBS to achieve a concentration of 3 × 10^5^ cells mL^−1^. Single‐cell isolation and mRNA capture were performed using the Singleron Matrix Single Cell Processing System (Singleron, China). Subsequently, scRNA‐seq libraries were constructed through reverse transcription, amplification, and library preparation using the GEXSCOPE Single Cell RNA Library Kit (Singleron, China). Only libraries meeting quality standards underwent sequencing on the Illumina HiSeq 6000 platform (150 bp paired‐end reads).

### Single‐Cell RNA Sequencing Data Analysis

The raw single‐cell RNA sequencing data underwent processing using the CeleScope pipeline (version 1.12.0), with default parameters. This comprehensive pipeline encompasses quality control of reads, mapping, extraction of unique molecular identifiers (UMIs) and barcodes, as well as the generation of an expression matrix consisting of UMI counts. The details of this processing approach can be found in the CeleScope GitHub repository (https://github.com/singleron‐RD/CeleScope/).

Utilizing the UMI matrix derived from the four single‐cell libraries, the data underwent subsequent merging and filtering using Seurat (v4.3.0) in R (v4.1.3), according to the following criteria: genes detected in fewer than 3 cells, cells with fewer than 200 genes detected, cells with more than 5000 genes detected, cells with over 15% of UMI reads mapped to mitochondrial genes, and cells with a number of detected genes or UMIs surpassing the 98th percentile among all cells in the dataset.^[^ ^32^ ^]^ Then gene expression data underwent normalization using the LogNormalize method through the NormalizeData function. The top 2000 variable genes were identified with the FindVariable function. Subsequent data scaling was achieved using the ScaleData function, followed by principal component analysis (PCA). To enhance integration across the four samples, the Harmony algorithm was applied. UMAP dimensionality reduction and cell clustering were then performed using Seurat's FindNeighbors and FindClusters functions.

### Cell–Cell Interaction Analysis

To uncover potential effects of immunotherapy on cellular communications across different cell clusters, it employed Cellchat to identify significant cell–cell interactions, with ligand‐receptor pairs exhibiting a p‐value < 0.05 considered as significant interactions.^[^ ^33^ ^]^ This analysis was carried out separately for each group, and the results were integrated for comparative analysis.

### Trajectory Inference of Fibroblast Cells and CD4^+^ T Cells

The cell differentiation trajectory of fibroblast cell populations was reconstructed utilizing the Monocle2 algorithm (v2.28).^[^ ^14^ ^]^ After isolating the expression matrix of fibroblast cell clusters from the overall Seurat object, it randomly sampled 5000 qualified cells for trajectory analysis. Genes expressed in fewer than 10 cells were excluded, and the top 2000 highly variable genes were identified using the “differentialGeneTest” function. These genes were subsequently utilized to order the cells in pseudotime analysis. For visualizing and interpreting the results with the plot_cell_trajectory function in Monocle2, dimensionality reduction was performed using the DDRTree method.

To infer the pseudotime trajectory of CD4^+^ T cells, the CD4^+^ T cell subtypes (including Tregs, CD4_Ccr7 and CD4_Ifng) were extracted and reclustered. After re‐clustering, the counts matrix of CD4^+^ T cells along with the UMAP results were then transferred into Monocle3 (v1.3.4). Subsequently, trajectories were constructed utilizing the learn_graph function, and the results were visualized using the plot_cells function.

### Prognostic Assessment of apCAF Score and its Correlation with Treg Score

The RNA‐seq and clinical data of The Cancer Genome Atlas (TCGA) pancreatic adenocarcinoma (PAAD) cohort were download from UCSC xena (https://xena.ucsc.edu/).^[^ ^23^ ^]^ 178 pancreatic adenocarcinoma patients with both available transcriptome data and clinical information were included. The apCAF and Treg scores for each tumor were calculated using ssGSEA, based on the top 20 marker genes with the highest fold change within the respective clusters from the pancreatic tumor single‐cell analysis (Table S2, Supporting Information). The relationship between apCAF score and Treg score was assessed using Spearman's rank correlation in the TCGA pancreatic cancer cohort and validated in another pancreatic tumor dataset (GSE224564).^[^ ^15^ ^]^ Patients were then stratified into two groups based on the median expression level of the apCAF score. Survival curves between the two groups were estimated using the Kaplan‐Meier method, and the significance was determined using the log‐rank test.

### Ethics Approval

The studies involving human‐derived samples were conducted in compliance with ethical regulations and approved by the Ethics Review Committee of Mengchao Hepatobiliary Hospital of Fujian Medical University (KESHEN 2021_100_02). All animal procedures were conducted according to the “National animal management regulations of China” and approved by the Animal Ethics Committee of Mengchao Hepatobiliary Hospital of Fujian Medical University (MCHH‐AEC‐2023‐12).

### Statistical Analysis

Statistical analysis of data was analyzed by one‐way or two‐way analysis of variance (ANOVA). *p < 0.05 was set as statistically significant, also including **p < 0.01, ***p < 0.001, ****p < 0.0001. Results were presented as mean ± standard error of the mean (SEM) at least three experiments by the software of GraphPad Prism (San Diego, USA).

## Conflict of Interest

The authors declare no conflict of interest.

## Author Contributions

Z.C., R.T. and X.L. conceived and designed the study. Z.C., Z.L., W.Z., F.L., X.D., H.Y., and Y.G. performed the experiments. Z.C., Z.L., W.Z., G.C., X.Y., H.Y., R.T. and X.L. analyzed and interpreted the data. Z.C., Z.L., R.T. and X.L. wrote and revised the manuscript. R.T. and X.L. supervised the study. All authors have approved the final version of the manuscript.

## Supporting information



Supporting Information

## Data Availability

The data that support the findings of this study are available from the corresponding author upon reasonable request.
