# Supplementary material for: Targeting Mesothelin Enhances Personalized Neoantigen Vaccine Induced Antitumor Immune Response in Orthotopic Pancreatic Cancer Mouse Models
Source: Adv Sci (Weinh). 2025 Jan 31;12(12):2407976. doi: 10.1002/advs.202407976 (PMC11948035; doi:10.1002/advs.202407976)
Supplement: Supplementary file 1 — Supporting Information [file ADVS-12-2407976-s001.docx]

**Targeting mesothelin enhances** **personalized neoantigen vaccine induced antitumor immune response in** **orthotopic pancreatic cancer mouse models**

Zhixiong Cai^1,2,3,#^, Zhenli Li^1,2,3,#^, Wenting Zhong^1,2,3,#^, Fangzhou Lin^1,2,3^, Xiuqing Dong^1,2,3^, Honghao Ye^1,2,3^, Yutong Guo^1,2,3^, Geng Chen^1,2,3^, Xiaoling Yu^1,2,3^, Haijun Yu^4^, Ruijing Tang^1,2,3,*^, and Xiaolong Liu^1,2,3,*^

1. The United Innovation of Mengchao Hepatobiliary Technology Key Laboratory of Fujian Province, Mengchao Hepatobiliary Hospital of Fujian Medical University, Fuzhou, P. R. China;

2. The Liver Center of Fujian Province, Fujian Medical University, Fuzhou, P. R. China;

3. Mengchao Med-X Center, Fuzhou University, Fuzhou, P. R. China;

4. State Key Laboratory of Drug Research & Center of Pharmaceutics, Shanghai Institute of Materia Medica, Chinese Academy of Sciences, Shanghai, P. R.China;

#These authors contributed equally.

*Correspondence: Xiaolong Liu (xiaoloong.liu@gmail.com, Mengchao Hepatobiliary Hospital of Fujian Medical University, Fuzhou 350025, P. R. China); Ruijing Tang (trjtrjtrj@163.com, Mengchao Hepatobiliary Hospital of Fujian Medical University, Fuzhou 350025, P. R. China)

**Supplementary Information**


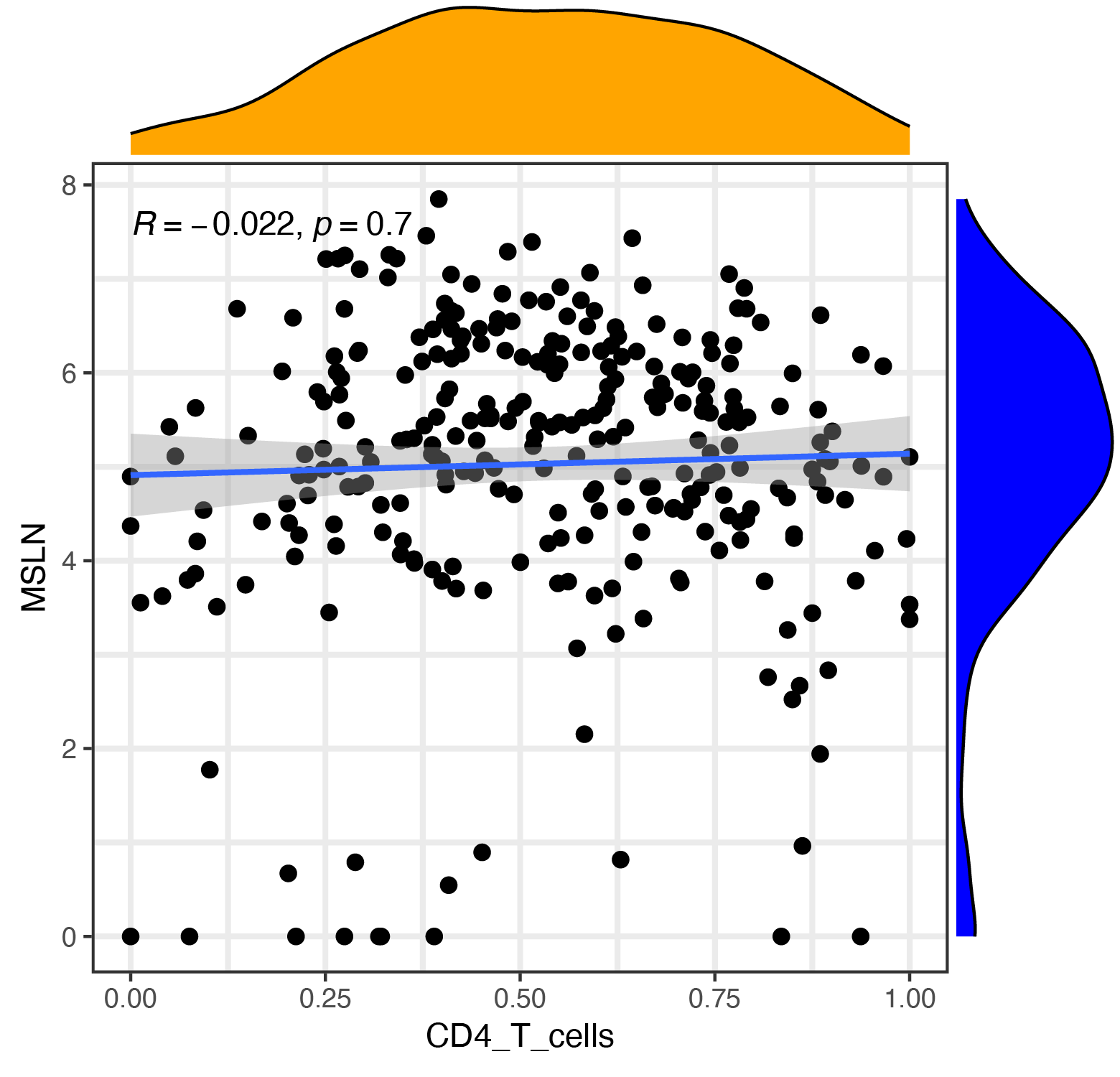


**Figure S1.** The correlation between MSLN expression and CD3^+^CD4^+^ T cells in 3 combined datasets.


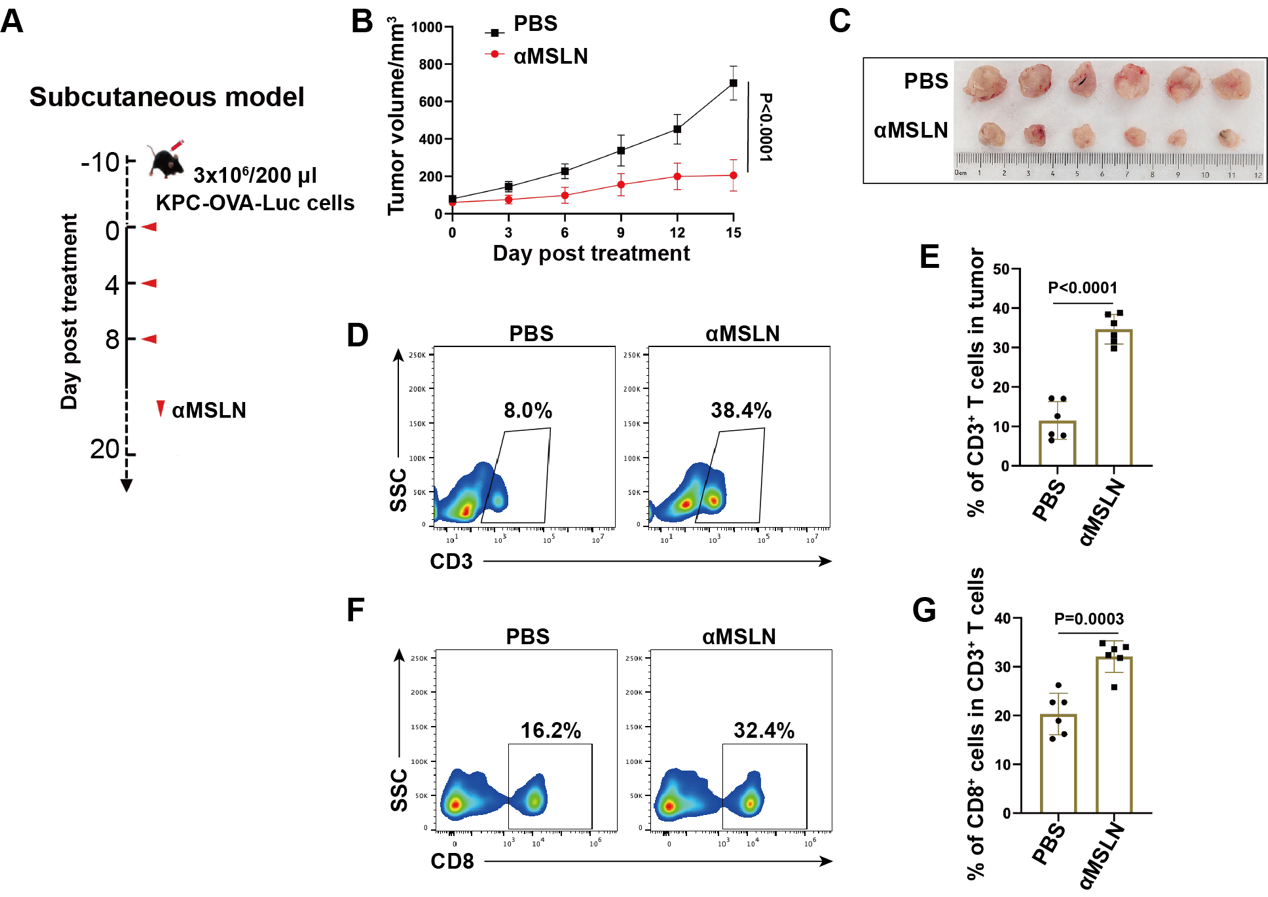


**Figure S2.** The antitumor effects of αMSLN in KPC-OVA-Luc tumor-bearing mice. A) Schematic diagram showing the timeline of subcutaneous pancreatic cancer model construction and treatment. B, C) Tumor volume monitoring in KPC-OVA-Luc tumor-bearing C57BL/6 mice (n=6) after receiving PBS and αMSLN treatment for three times, respectively. D-G) The percentage and statistic analysis of CD3^+^ and CD8^+^ T cells detected in KPC-OVA-Luc tumor after treatment (n=6) by flow cytometry.


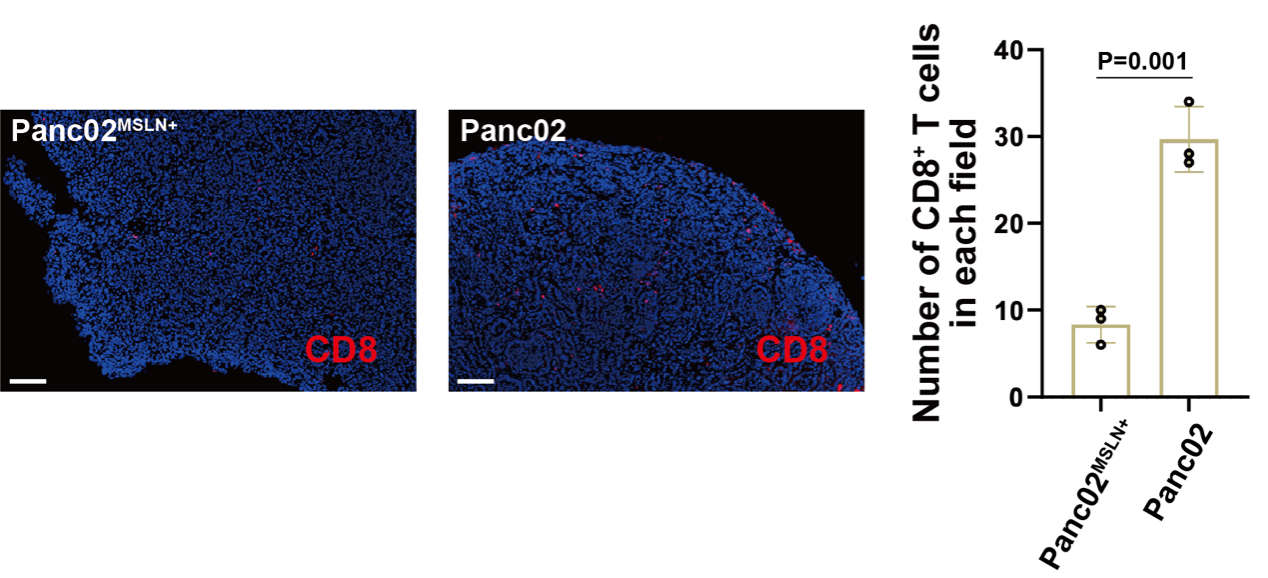


**Figure S3.** The represent graph and statistic analysis of the infiltration of CD8^+^ T cells by immune fluorescent staining. Scale bar = 100 µm. Panc02^MSLN+^ represents the MSLN-overexpressing cell line generated by lentiviral infection.


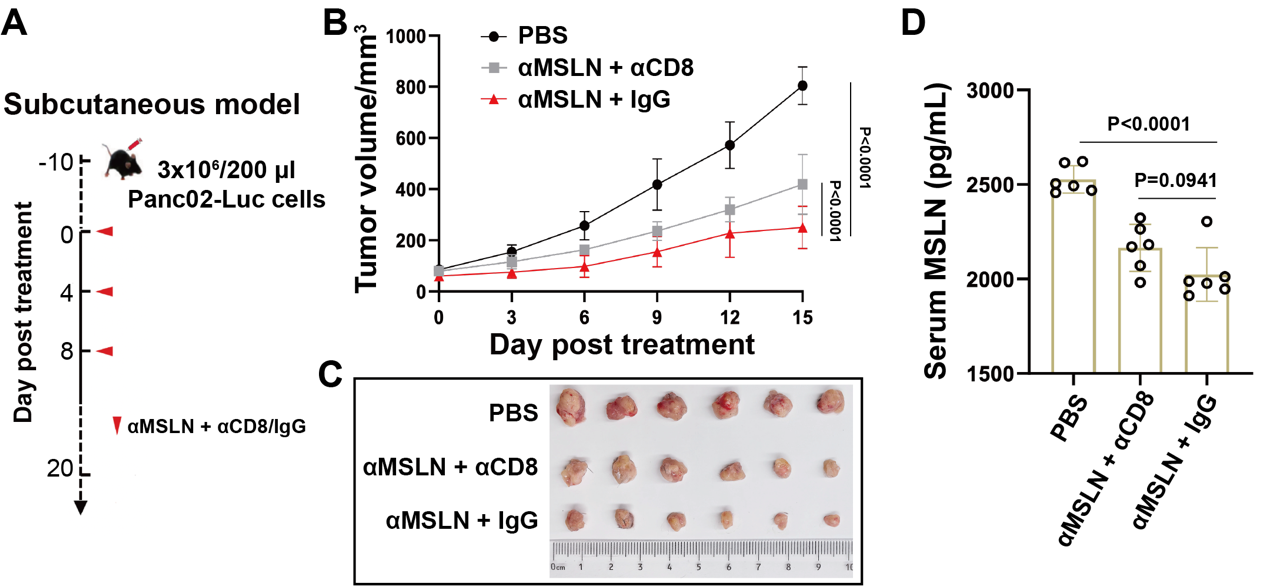


**Figure S4.** A) Schematic diagram showing the timeline of subcutaneous pancreatic cancer model construction and treatment. B, C) Tumor volume monitoring in Panc02 tumor-bearing C57BL/6 mice (n=6) after receiving PBS, αMSLN plus αCD8, and αMSLN plus IgG isotype treatment for three times, respectively. D) Quantification of soluble MSLN levels in the serum of mice bearing tumor under different treatment using ELISA (n=6).


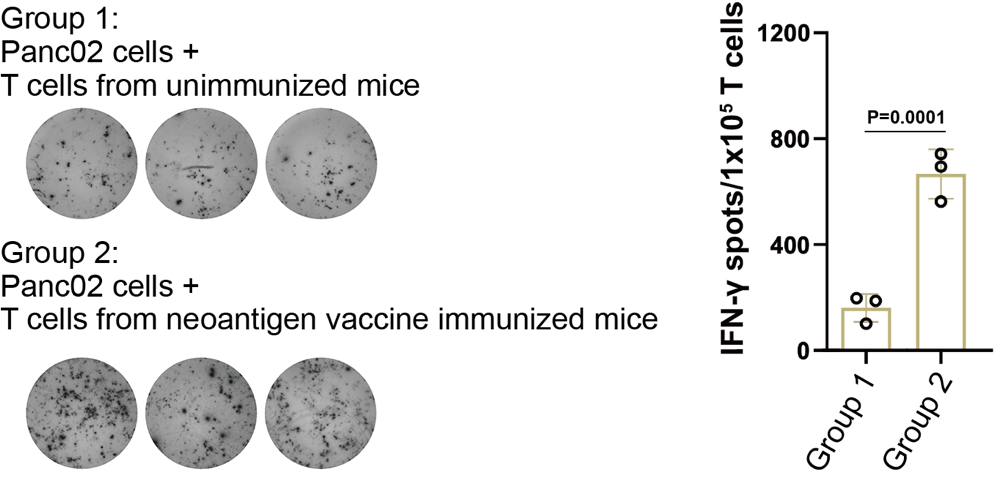


**Figure S5:** The spot assay of IFN-γ secretion by T cells after co-culturing with Panc02 cells.


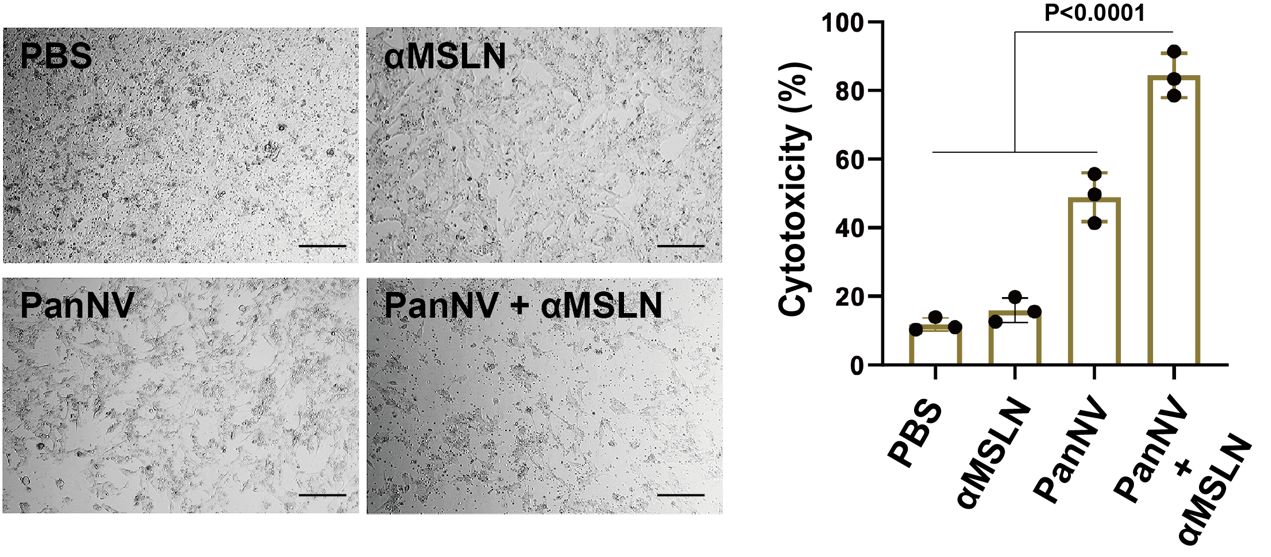


**Figure S6:** In vitro cytotoxicity analysis induced by CD8^+^CD69^+^ T cells against Panc02 cells determined by LDH assay (n=3). Scale bar = 100 µm.


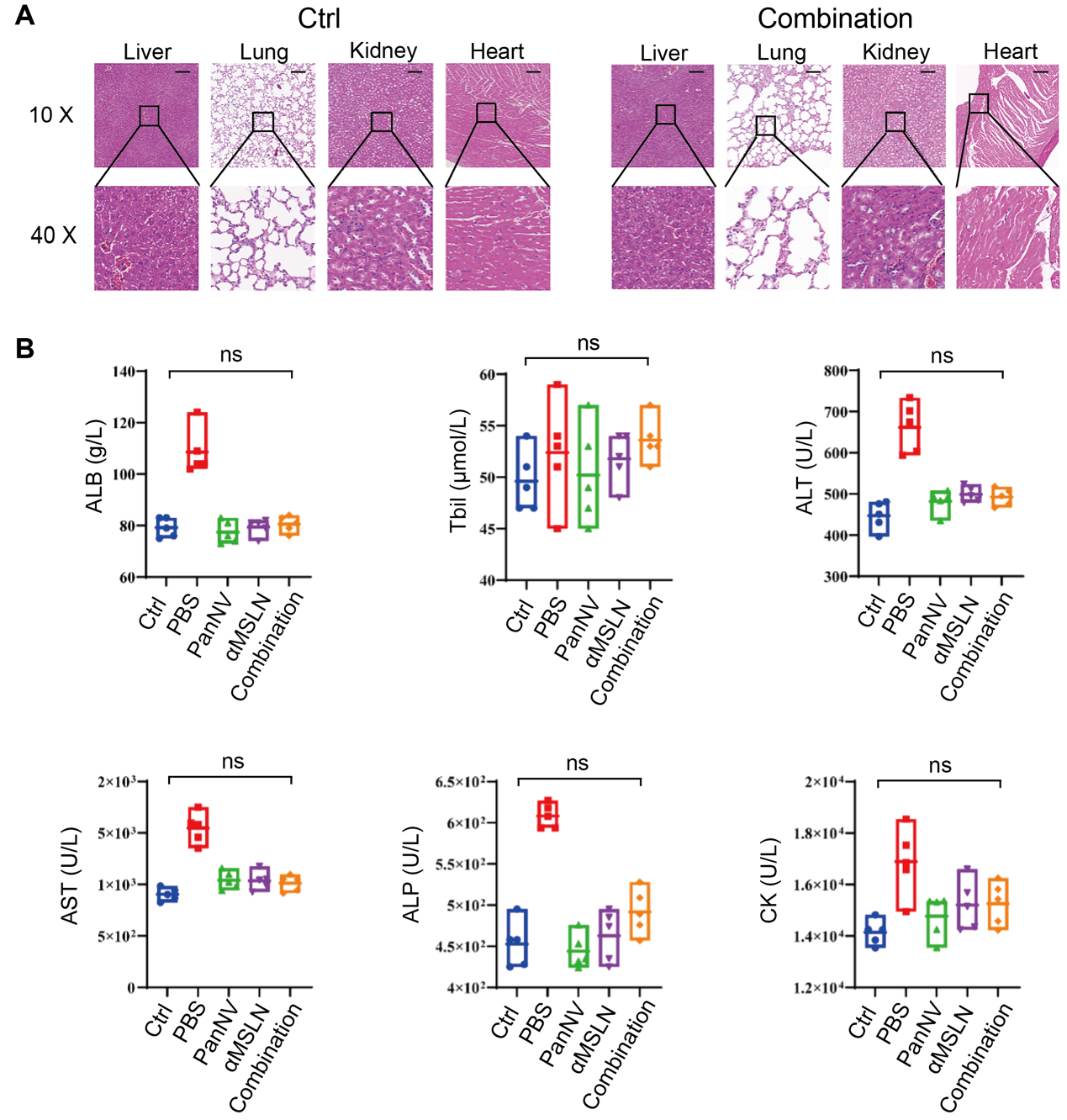


**Figure S7.** Safety assessment of the combined treatment of PanNV plus αMSLN in orthotopic pancreatic cancer. A) H&E staining of major organs from Panc02 bearing mice after receiving different treatment as indicated. Scale bar, 100 μm. B) The changes of biochemical indicators in mice receiving different treatments as indicated.

**
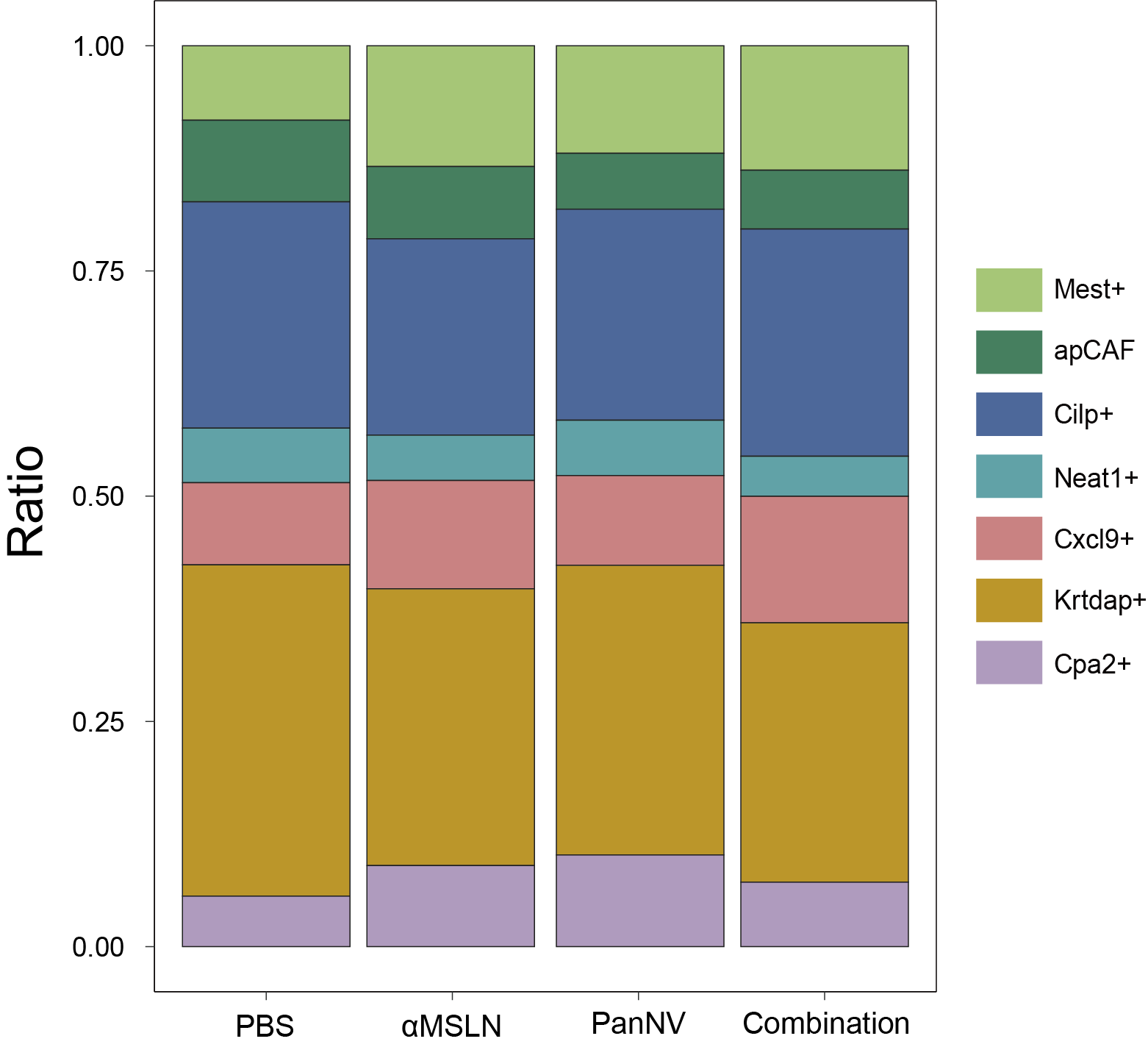
**

**Figure S8.** Proportion of each fibroblast subtype across all four groups.


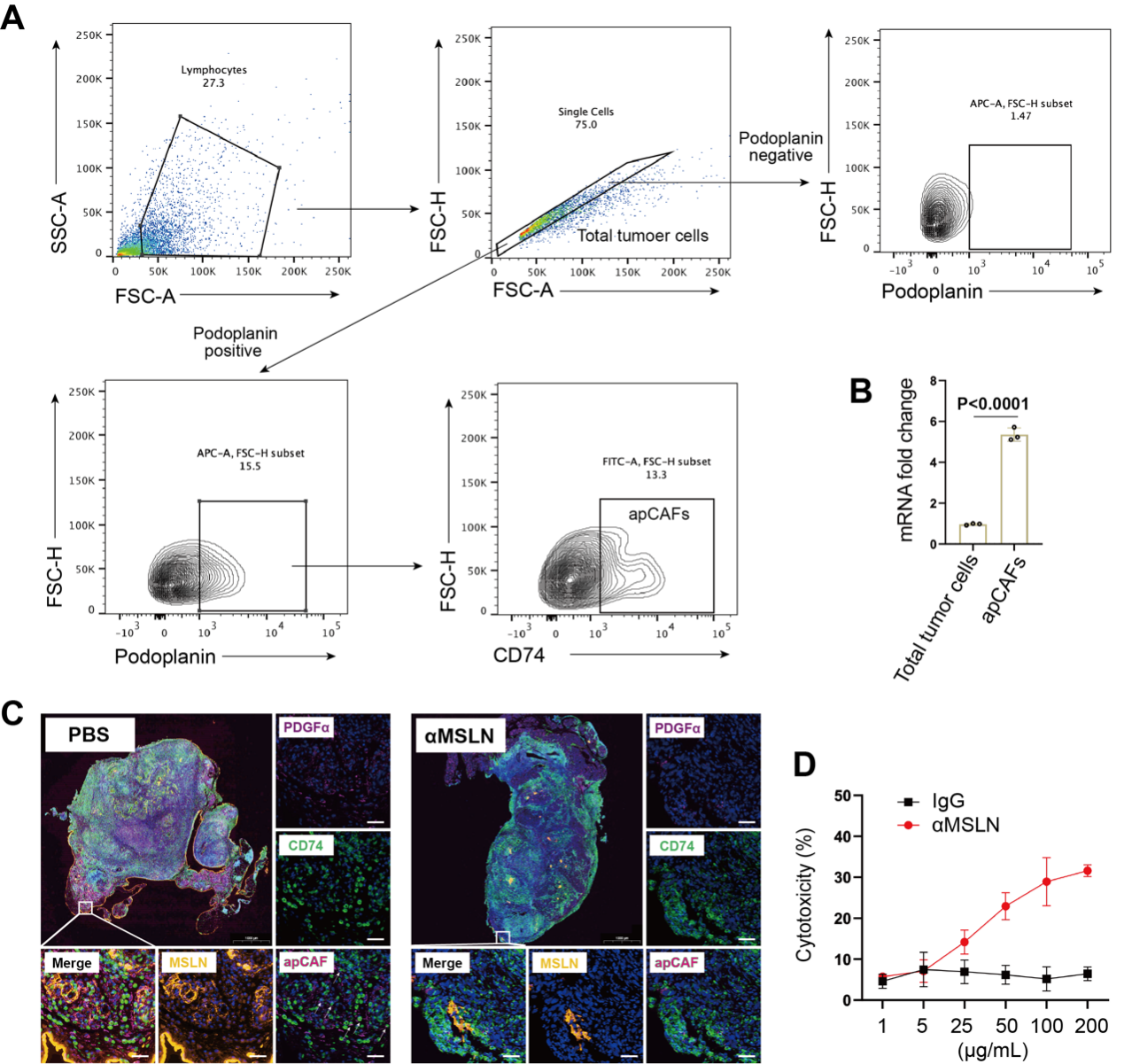


**Figure S9.** A) The sorting strategy for apCAF based on the two markers, Podoplanin and CD74. B) qPCR analysis of the relative mRNA expression levels of MSLN in total tumor cells and apCAFs. C) Representative graph showing the expression of MSLN, apCAF, CD74, and PDGFα, visualized through multiplex immunofluorescent staining. Scale bar = 100 µm. D) apCAFs were used as target cells and treated with varying concentrations (1, 5, 25, 50, 100, or 200 µg/mL) of αMSLN in the presence of mouse spleen-derived effector cells at a 1:4 ratio. ADCC activity was assessed by measuring LDH release.


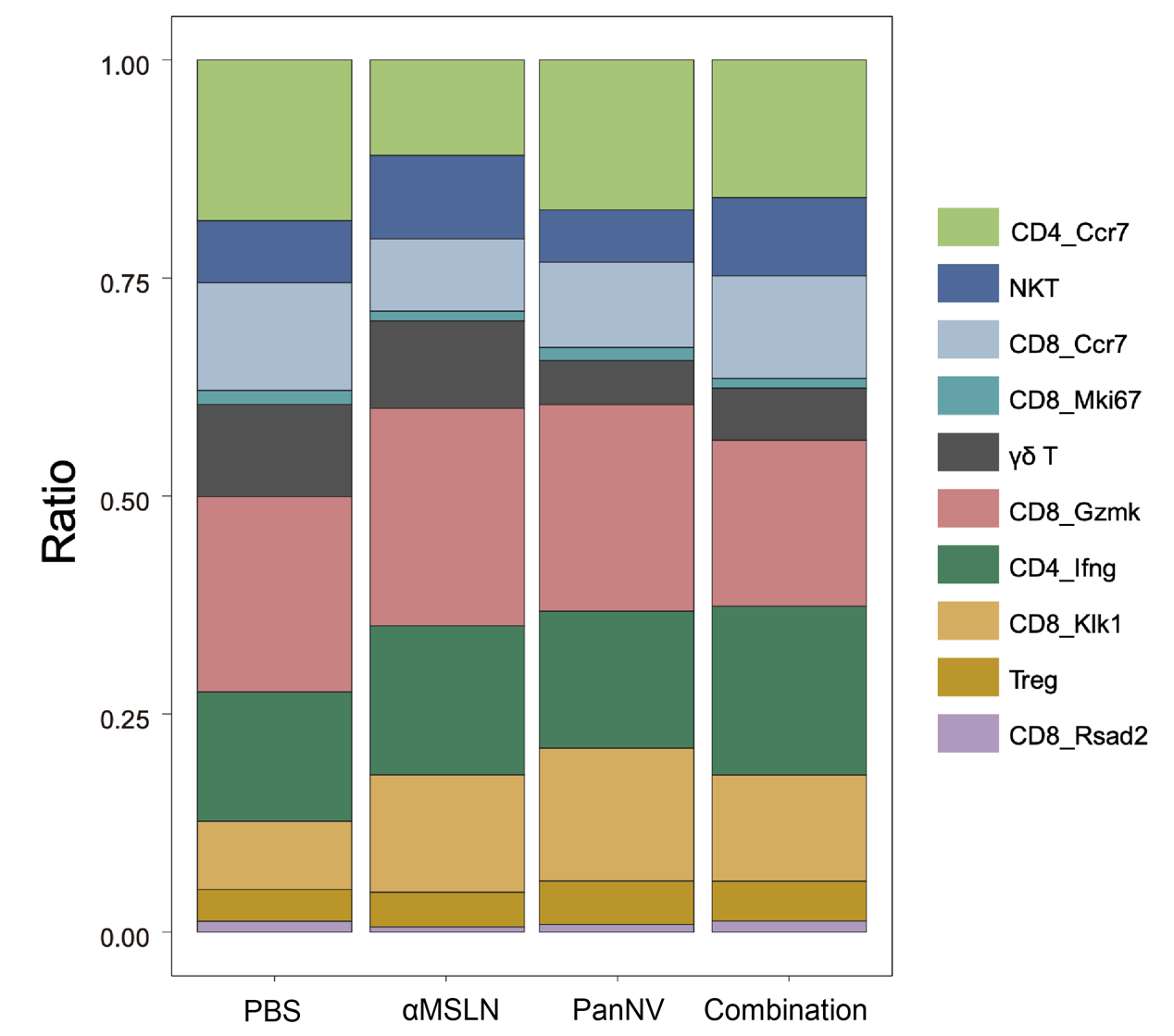


**Figure S10.** Proportion of each tumor infiltrated T cell subtype across all four groups.


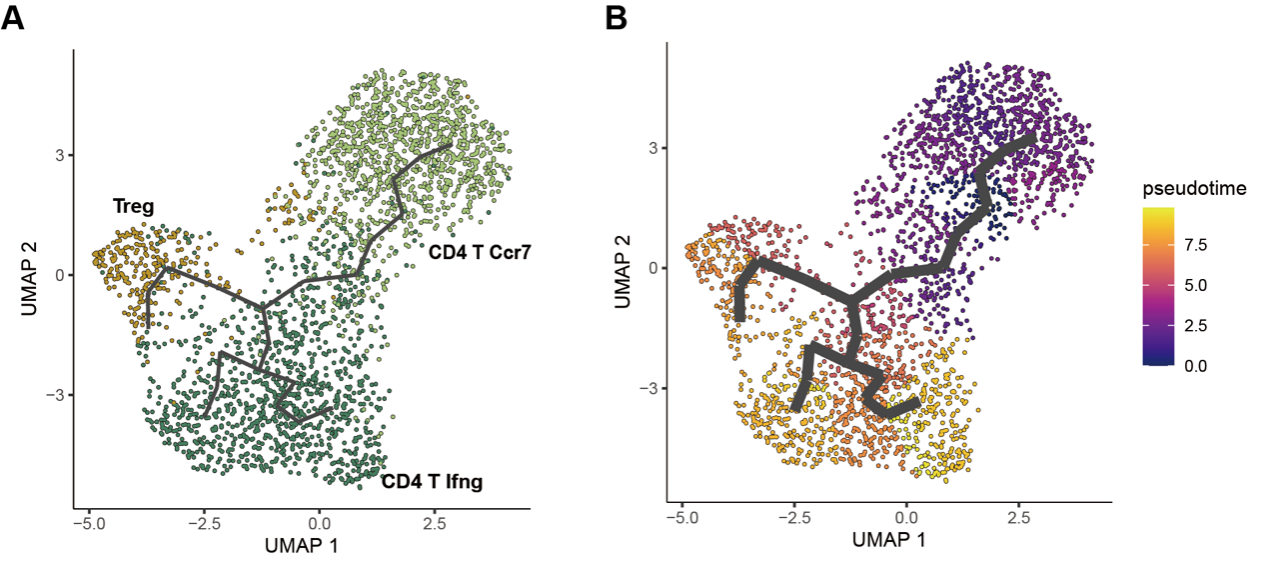


**Figure S11.** Pseudotime trajectory of CD4^+^ T cells. Pseudotime trajectory (a) colored by CD4^+^ T cell subtypes and (b) colored by pseudotime.


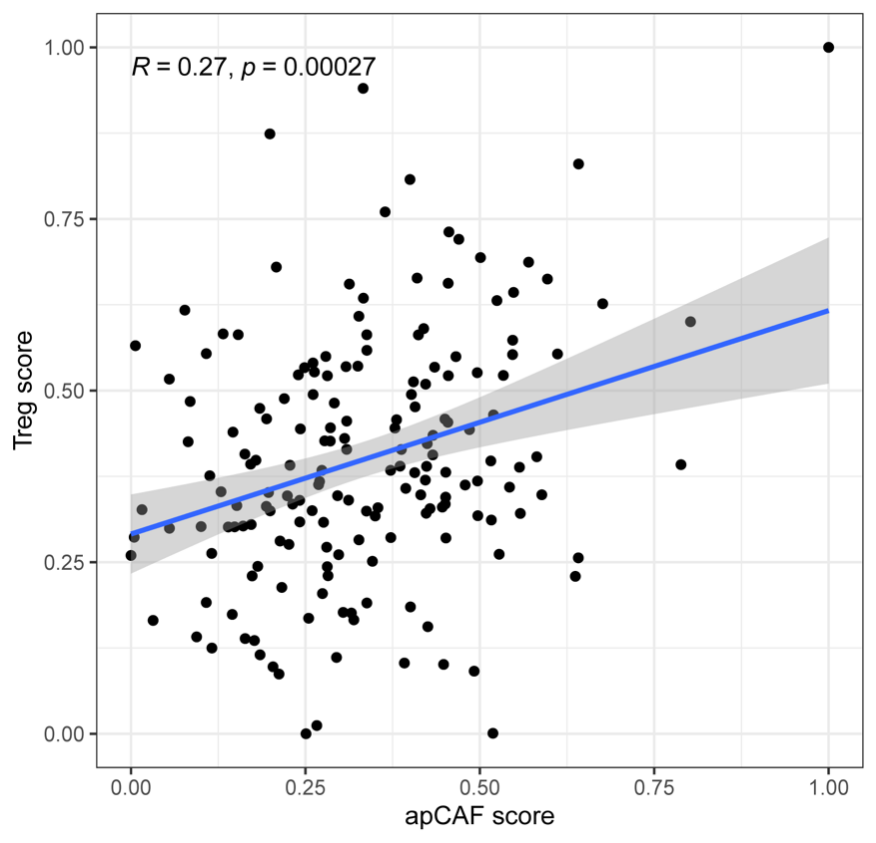


**Figure S12:** The correlation between apCAF score and Treg score in GSE224564 dataset.

| **Table S1.** Information of potential neoantigen mutations derived from Panc02 cell line | | | | | | | |
| --- | --- | --- | --- | --- | --- | --- | --- |
| **Chr** | **Position** | **Ref** | **Alt** | **Gene** | **Amino acid**  **substitution** | **Expression level^a^** | **Peptide^b^** |
| chrX | 73680521 | A | G | Slc6a8 | N543S | 40.71 | VCMGIFIFSIVYYEPLV |
| chr5 | 143478447 | G | T | Daglb | G238C | 5.32 | LVPSDIAACFTLLHQQQ |
| chr19 | 23228959 | C | A | Smc5 | V744F | 41.82 | STKIKEINFQKAKLVTE |
| chr11 | 116600110 | C | G | Rhbdf2 | G672A | 12.84 | ISIIFILSAITGNLASA |
| chr11 | 103377425 | C | G | Plekhm1 | E573D | 10.57 | GIWKEFFCDLSPLEFRL |
| chr4 | 16132815 | T | A | Ripk2 | T301S | 4.95 | VLRTFEDISFLEAVIQL |
| chr11 | 70218956 | G | T | Slc16a13 | H240N | 36.09 | IPYVHLVANLQDLGWDP |
| chr16 | 13122155 | G | T | Ercc4 | R183L | 12.22 | FCHVERVMLNLFVRKLY |
| chr11 | 102851769 | C | A | Eftud2 | G424C | 139.08 | RLVCKKFFCEFTGFVDM |
| chr9 | 113986427 | C | G | Fbxl2 | G256A | 16.81 | TDASLTALALNCPRLQV |
| chrX | 75391666 | A | C | Fundc2 | K91N | 28.41 | LANHTGYINVDWQRVEK |
| chr11 | 118411110 | C | A | Cant1 | W127L | 51.34 | SRAQEENTLFSYLKKGY |
| chr2 | 120906944 | T | C | Ubr1 | M1040V | 18.62 | HRQKIMAQVSALQKNFI |
| chr9 | 49028206 | G | T | Usp28 | W631L | 8.52 | DISVTESSLEELERDSY |
| chr15 | 100169290 | G | C | Dip2b | D558H | 9.57 | GETVVNVLHFKKDAGLW |
| chr2 | 25051725 | G | T | Pnpla7 | W1153C | 7.11 | GWWLLWKRCNPLATKVK |
| chr15 | 98125587 | G | T | Pfkm | A389S | 47.94 | NWEVYKLLSHVRPPVSK |
| chr2 | 121189374 | C | G | Tubgcp4 | A395G | 49.82 | VTEHDVNVGFQQSAHKV |
| chr9 | 20438211 | A | C | Zfp26 | F352L | 13.91 | YECKKCEKLFTHPVYLN |

a: Expression level (TPM) of the gene containing the neoantigen mutation.

b: The 17-mer mutated peptide with the mutated amino acid located in the middle.

**Table S2.** The top 20 marker genes for ssGSEA analysis

| **apCAF score** | **Treg score** |
| --- | --- |
| SLPI | FOXP3 |
| UPK3B | NEB |
| KRT19 | TNFRSF4 |
| CLU | IKZF2 |
| NKAIN4 | KLRG1 |
| GPM6A | CTLA4 |
| LGALS7 | IZUMO1R |
| CAV1 | TNFRSF9 |
| LRRN4 | IL2RA |
| MSLN | TNFRSF18 |
| UPK1B | ARL5A |
| HSPB1 | GLRX |
| GAS6 | CAPG |
| PKHD1L1 | CD2 |
| FGF1 | SAMSN1 |
| RSPO1 | MAF |
| TMEM176B | PIM1 |
| MUC16 | ICOS |
| CAVIN2 | NFKBIA |
| CD74 | VPS54 |
